# Supplementary figures and images for: Functional requirements for a Samd14-capping protein complex in stress erythropoiesis
Source: eLife. 2022 Jun 17;11:e76497. doi: 10.7554/eLife.76497 (PMC9282853; doi:10.7554/eLife.76497)

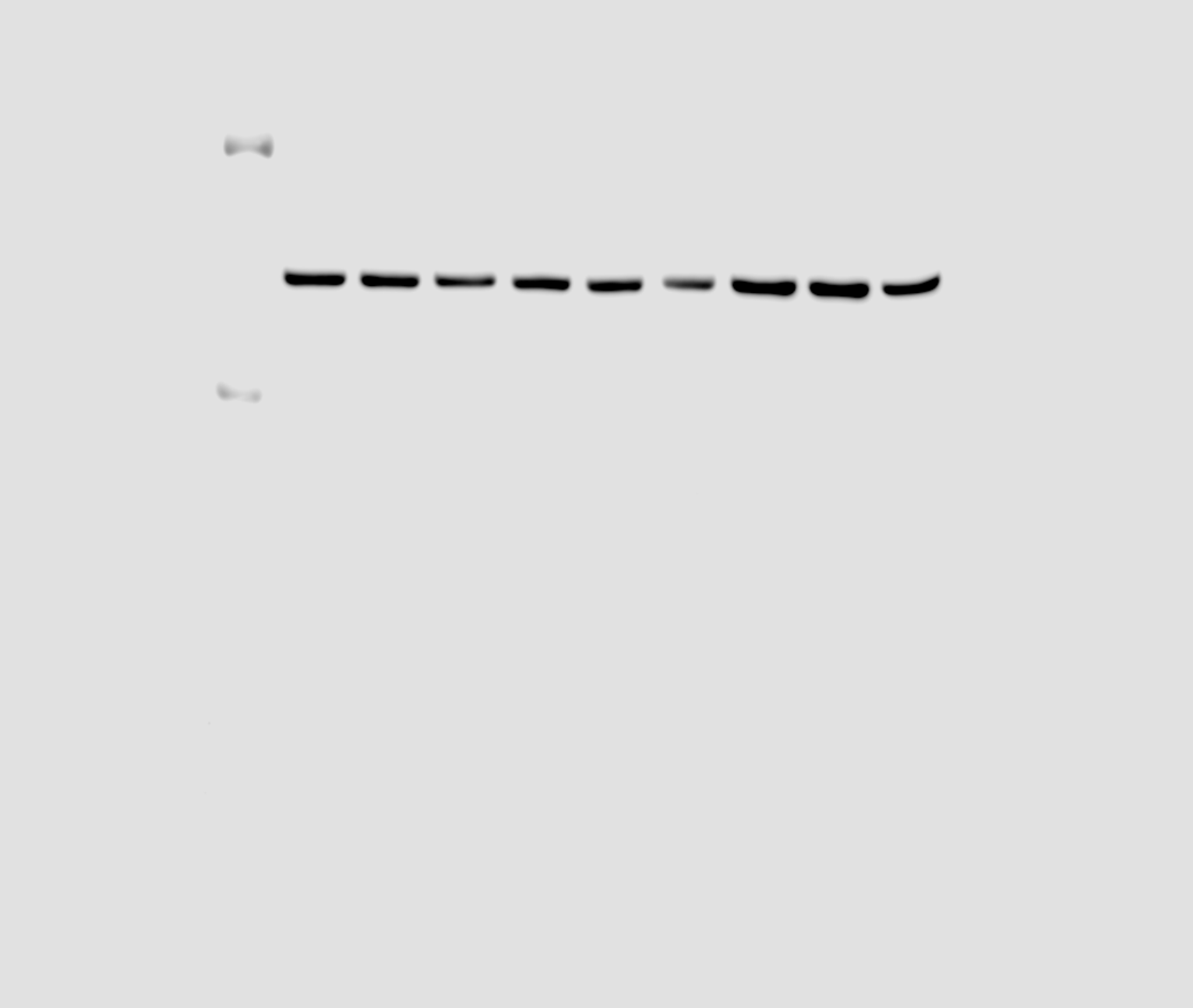

Supplement: Figure 1—source data 1. [file elife-76497-fig1-data1.zip › Figure 1-source data 1/Figure 1B-actin.tif]

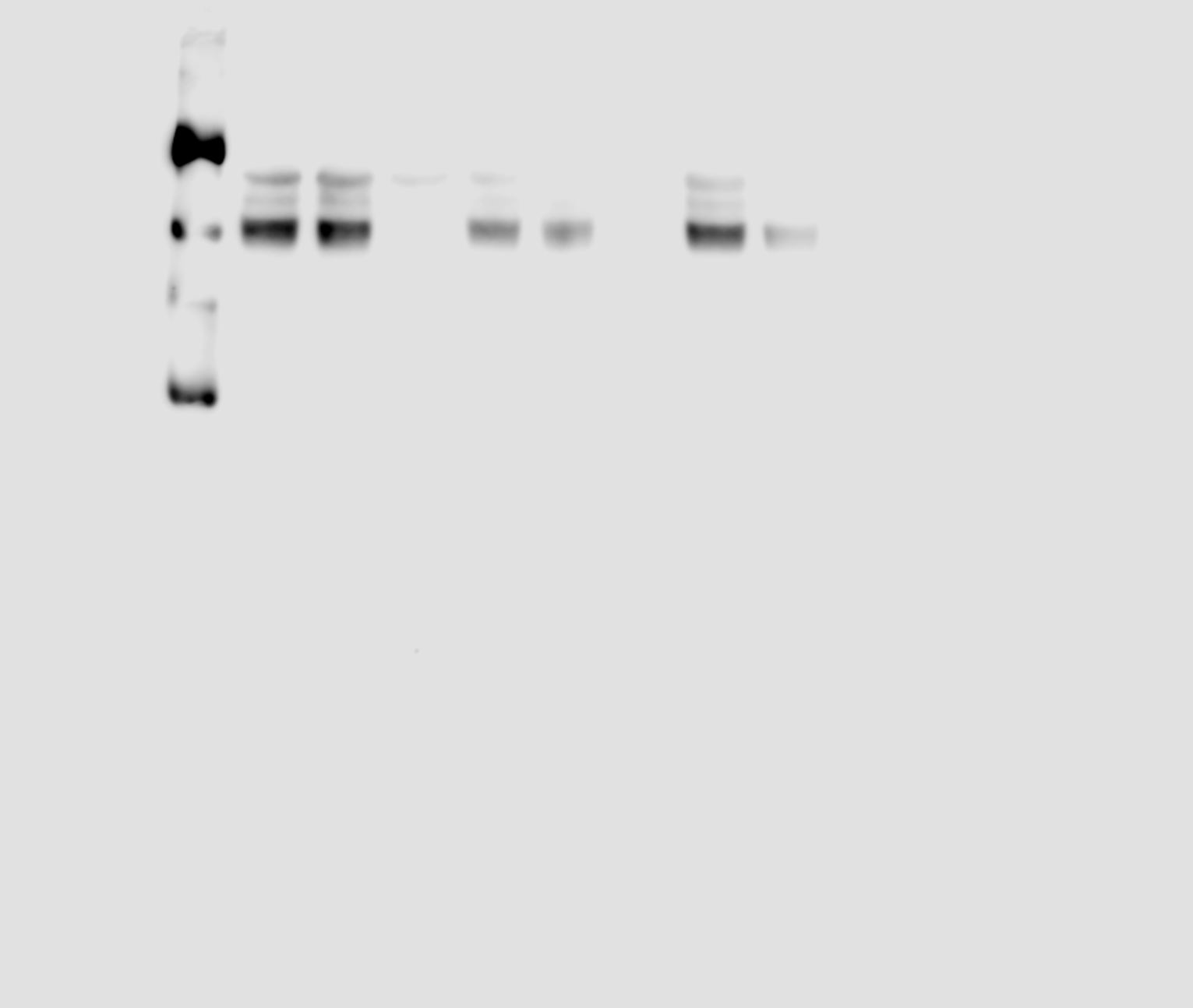

Supplement: Figure 1—source data 1. [file elife-76497-fig1-data1.zip › Figure 1-source data 1/Figure 1B-Samd14.tif]

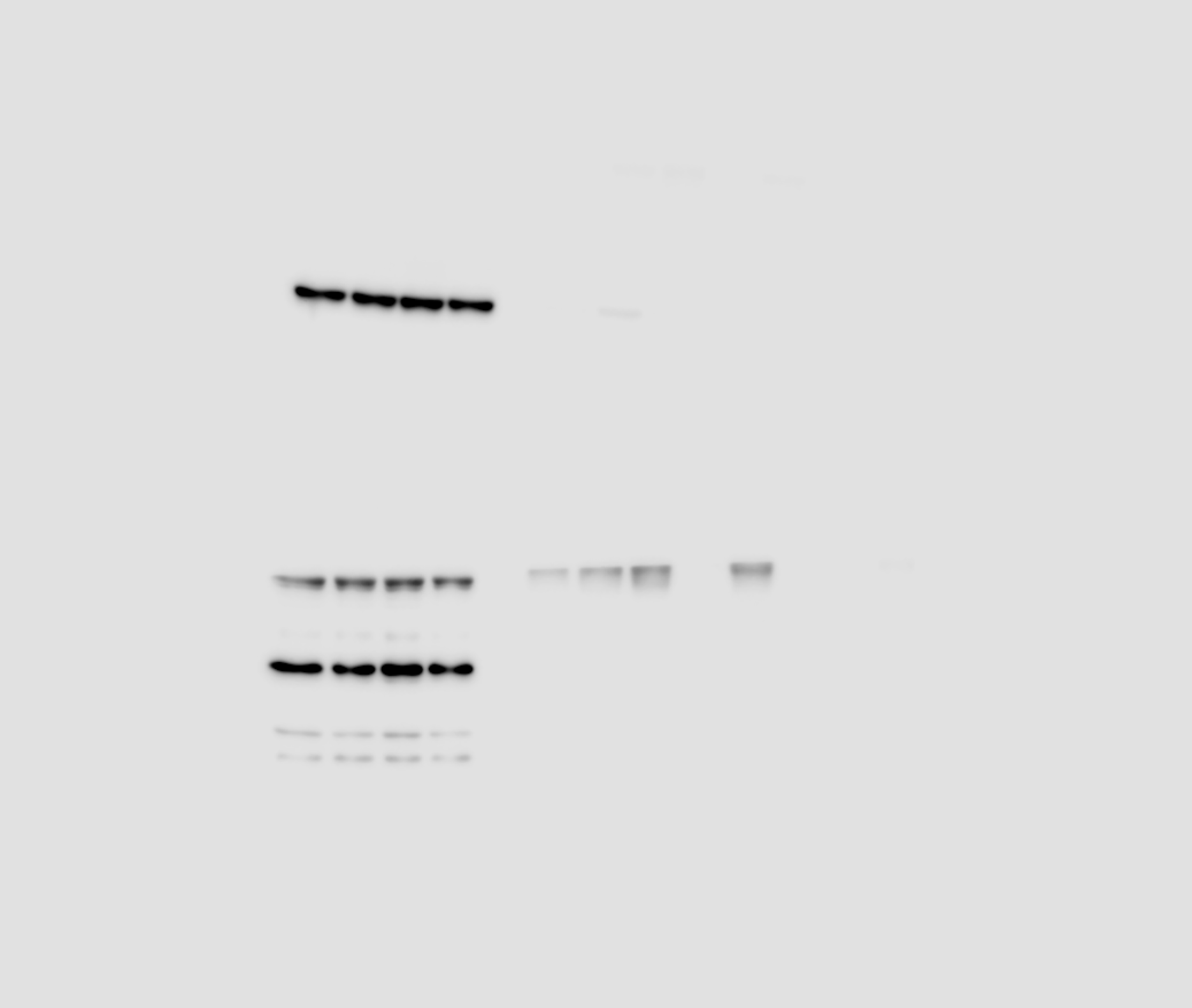

Supplement: Figure 1—source data 1. [file elife-76497-fig1-data1.zip › Figure 1-source data 1/Figure 1C-capzb Input.tif]

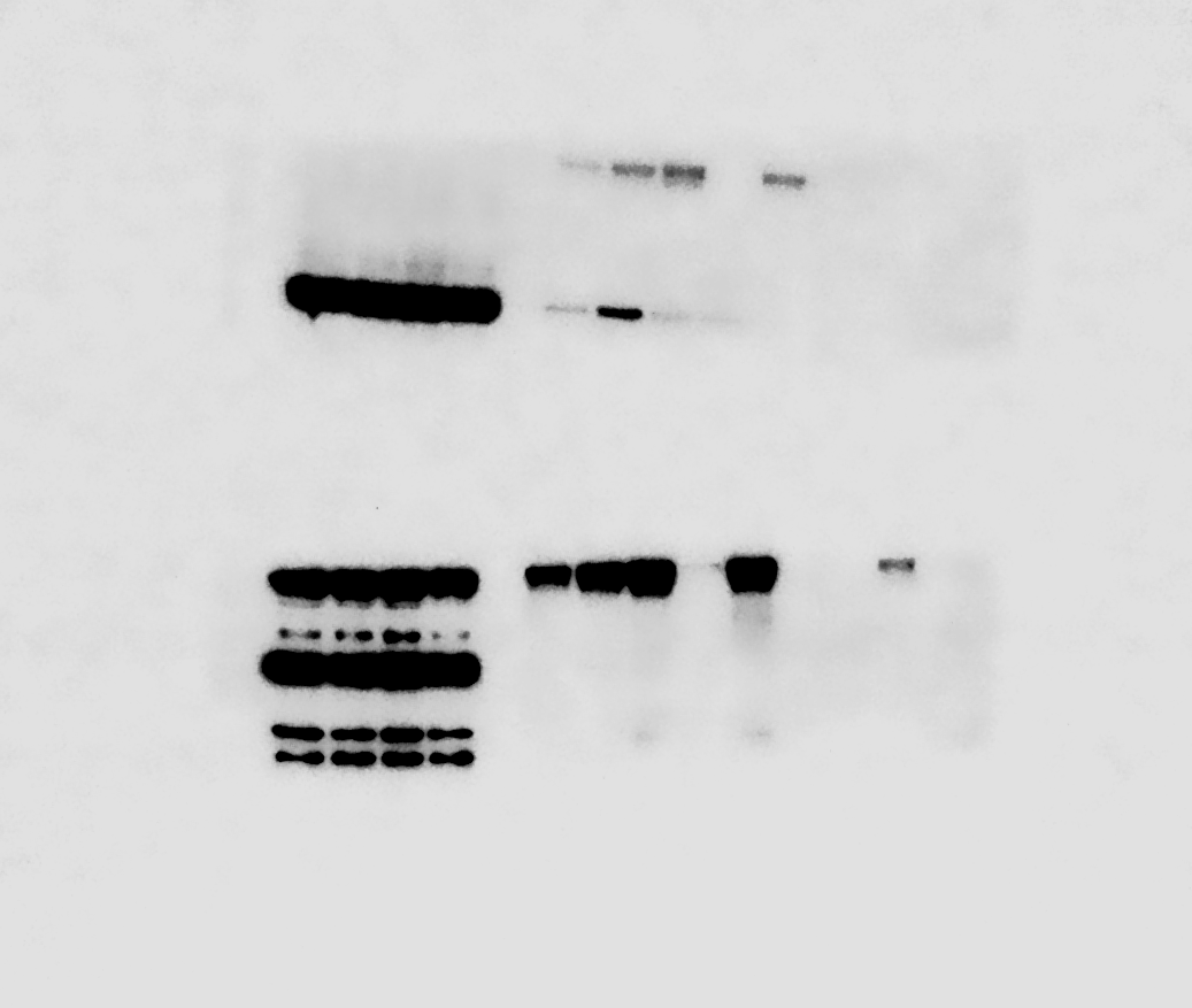

Supplement: Figure 1—source data 1. [file elife-76497-fig1-data1.zip › Figure 1-source data 1/Figure 1C-capzb IP.tif]

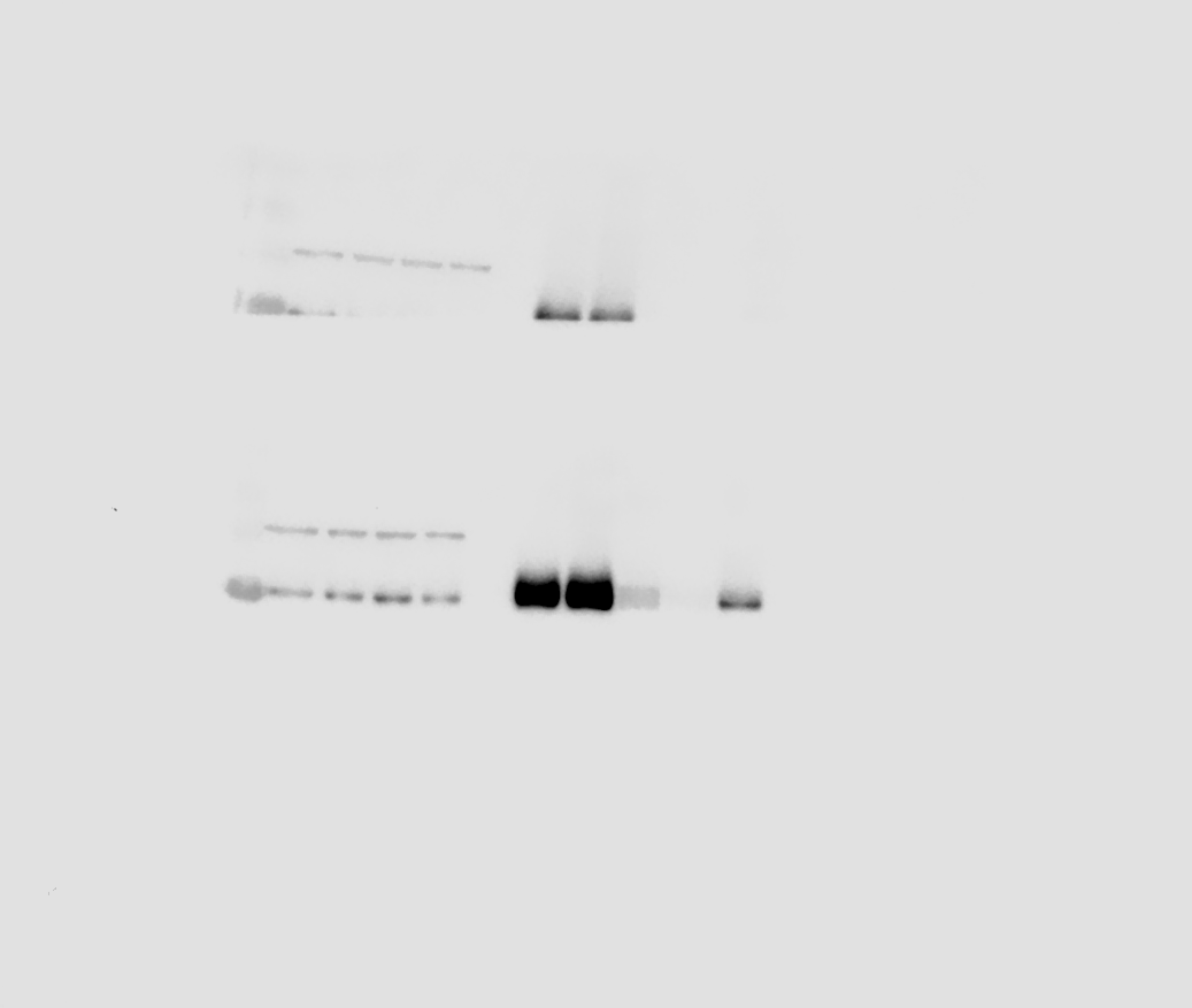

Supplement: Figure 1—source data 1. [file elife-76497-fig1-data1.zip › Figure 1-source data 1/Figure 1C-Samd14 Input and IP.tif]

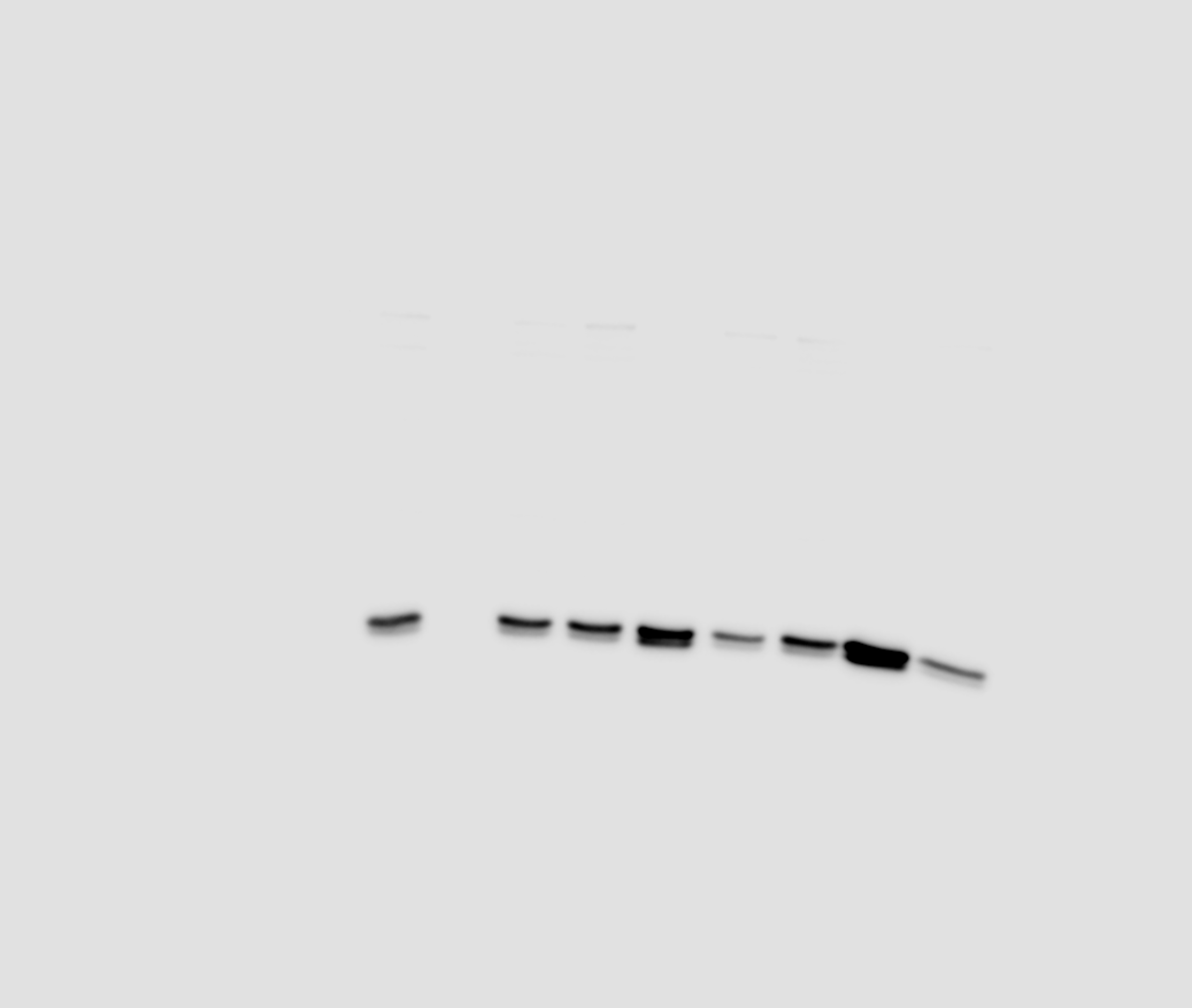

Supplement: Figure 1—source data 1. [file elife-76497-fig1-data1.zip › Figure 1-source data 1/Figure 1D-capza2.tif]

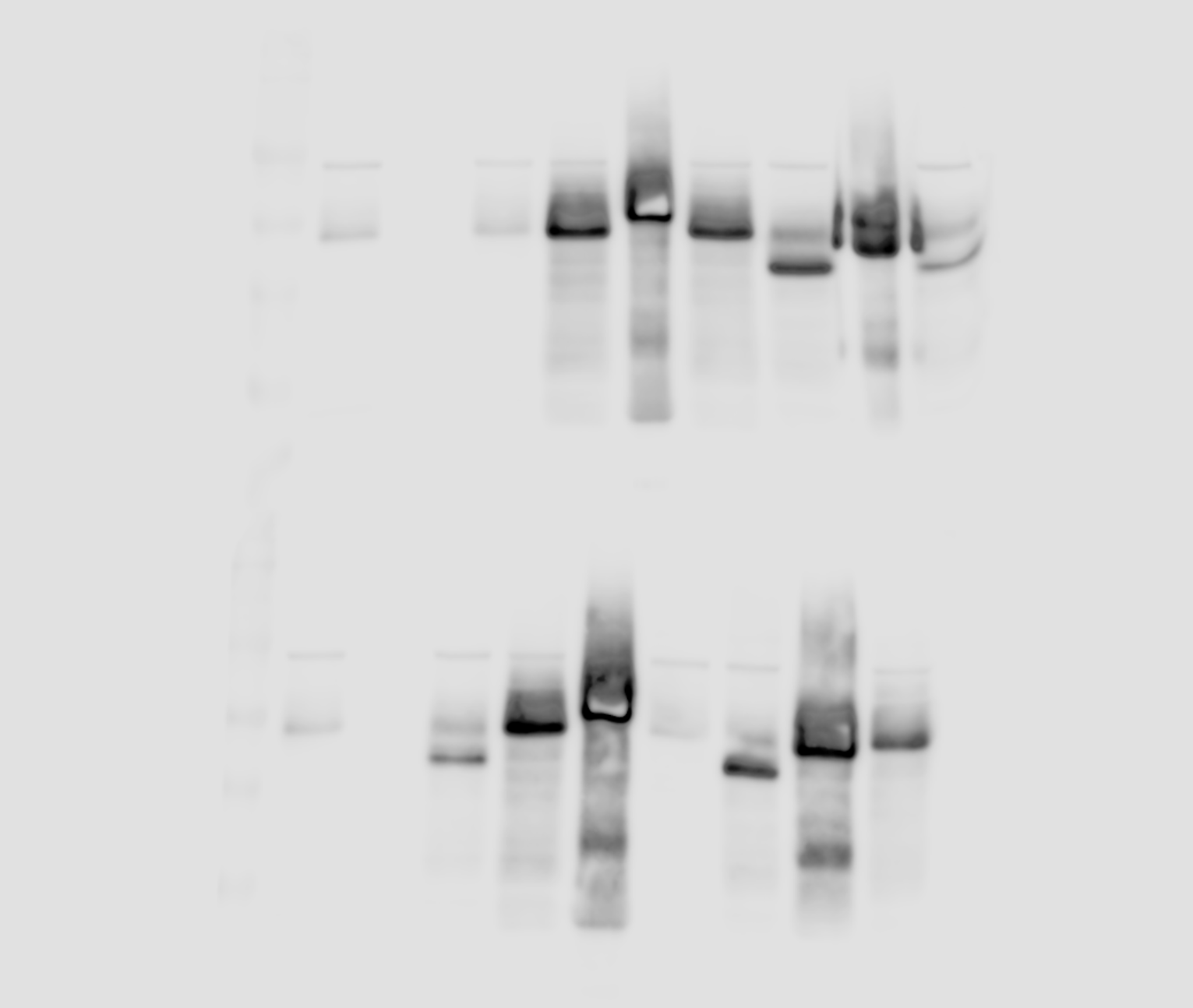

Supplement: Figure 1—source data 1. [file elife-76497-fig1-data1.zip › Figure 1-source data 1/Figure 1D-Samd14.tif]

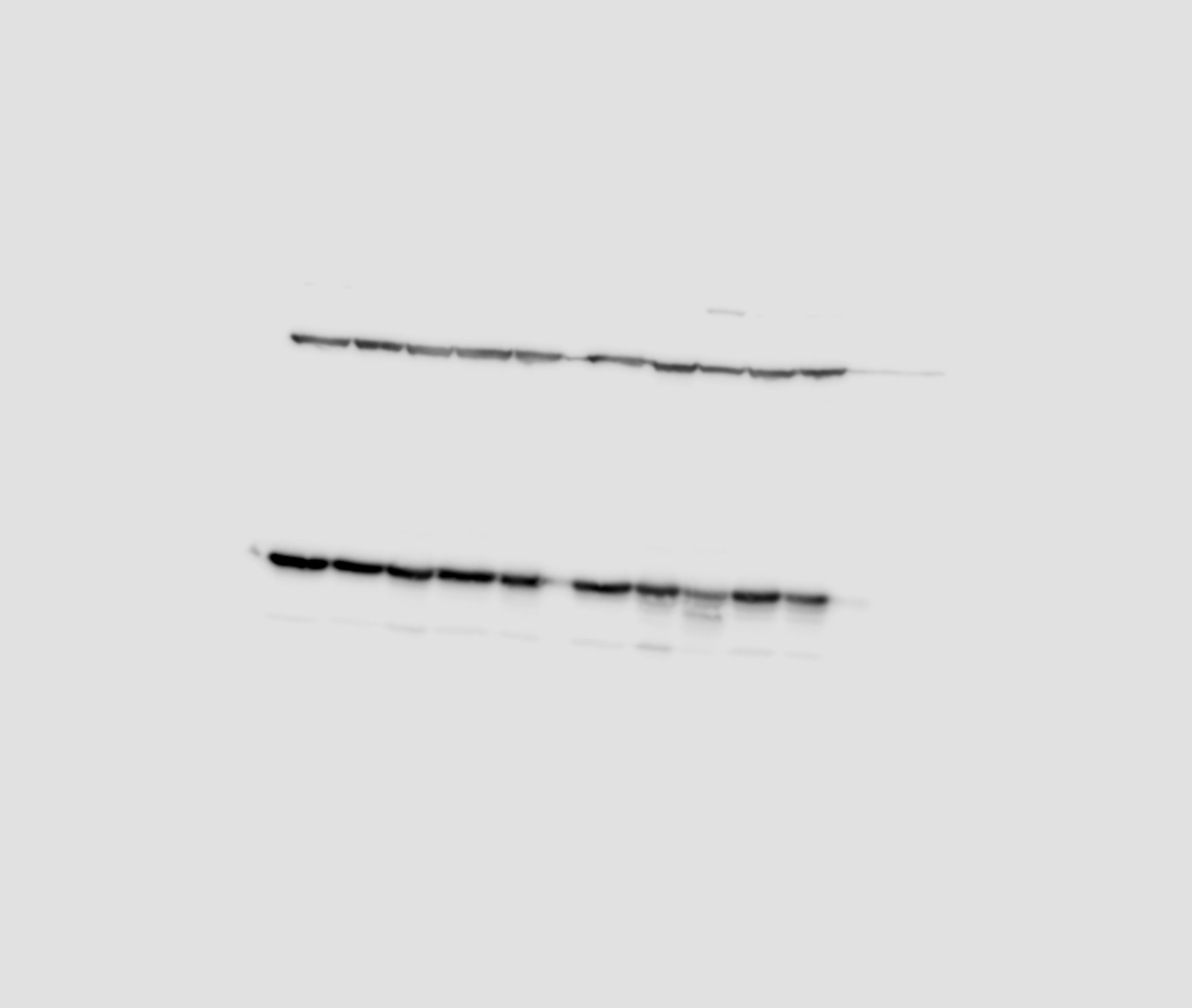

Supplement: Figure 1—source data 1. [file elife-76497-fig1-data1.zip › Figure 1-source data 1/Figure 1F-capzb and actin.tif]

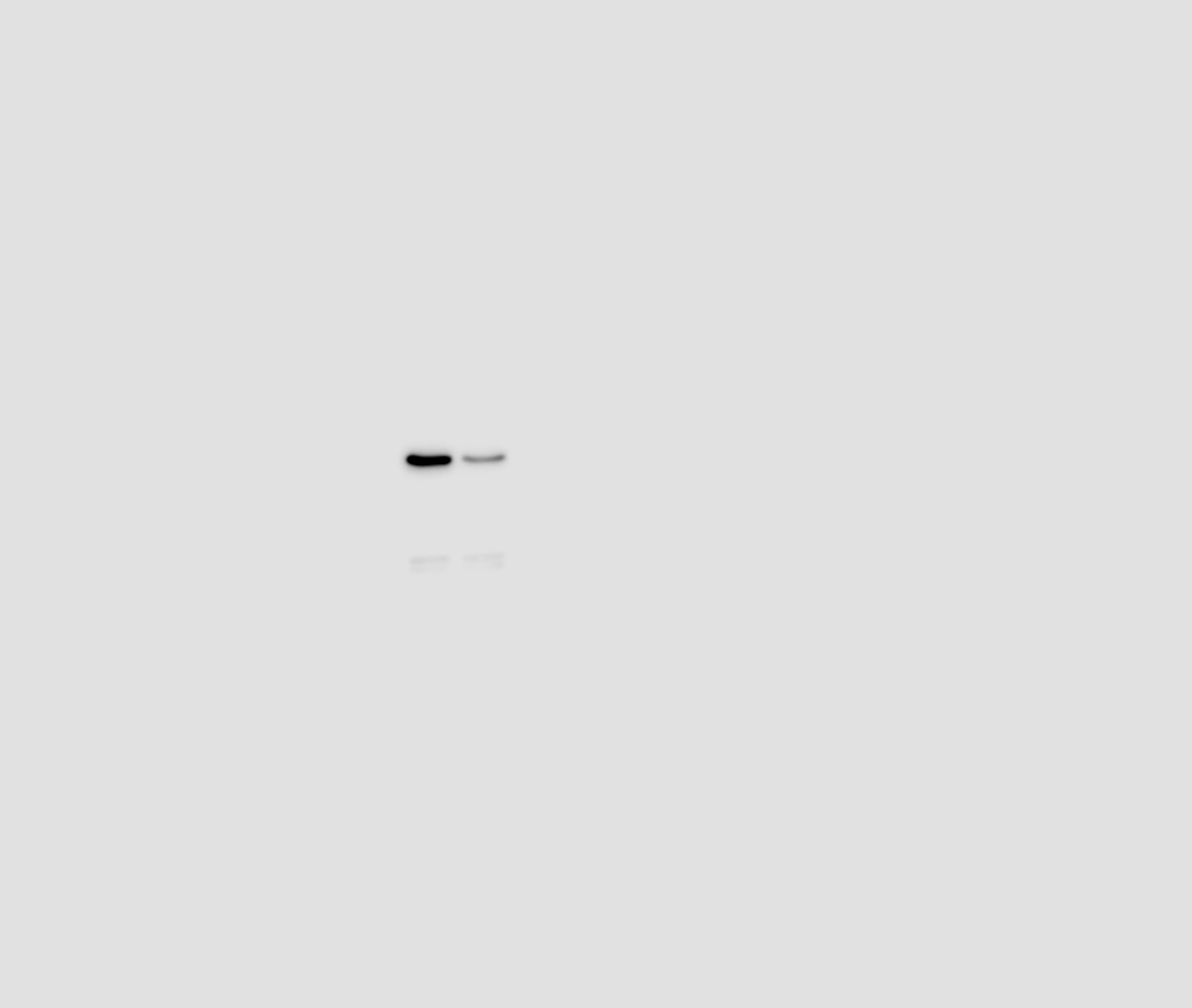

Supplement: Figure 2—source data 1. [file elife-76497-fig2-data1.zip › Figure 2-source data 1/Figure 2A capza1.tif]

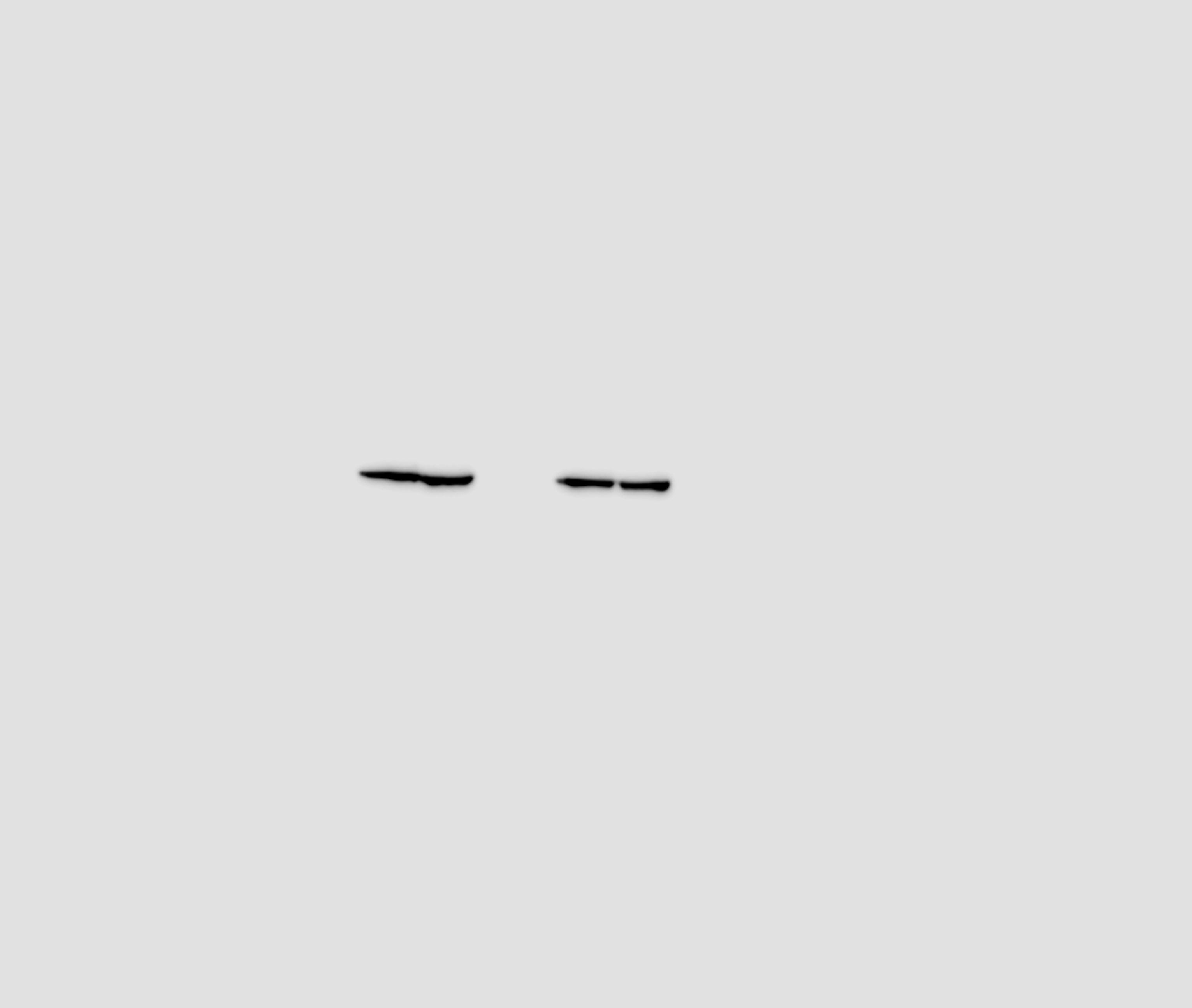

Supplement: Figure 2—source data 1. [file elife-76497-fig2-data1.zip › Figure 2-source data 1/Figure 2A Hsc70.tif]

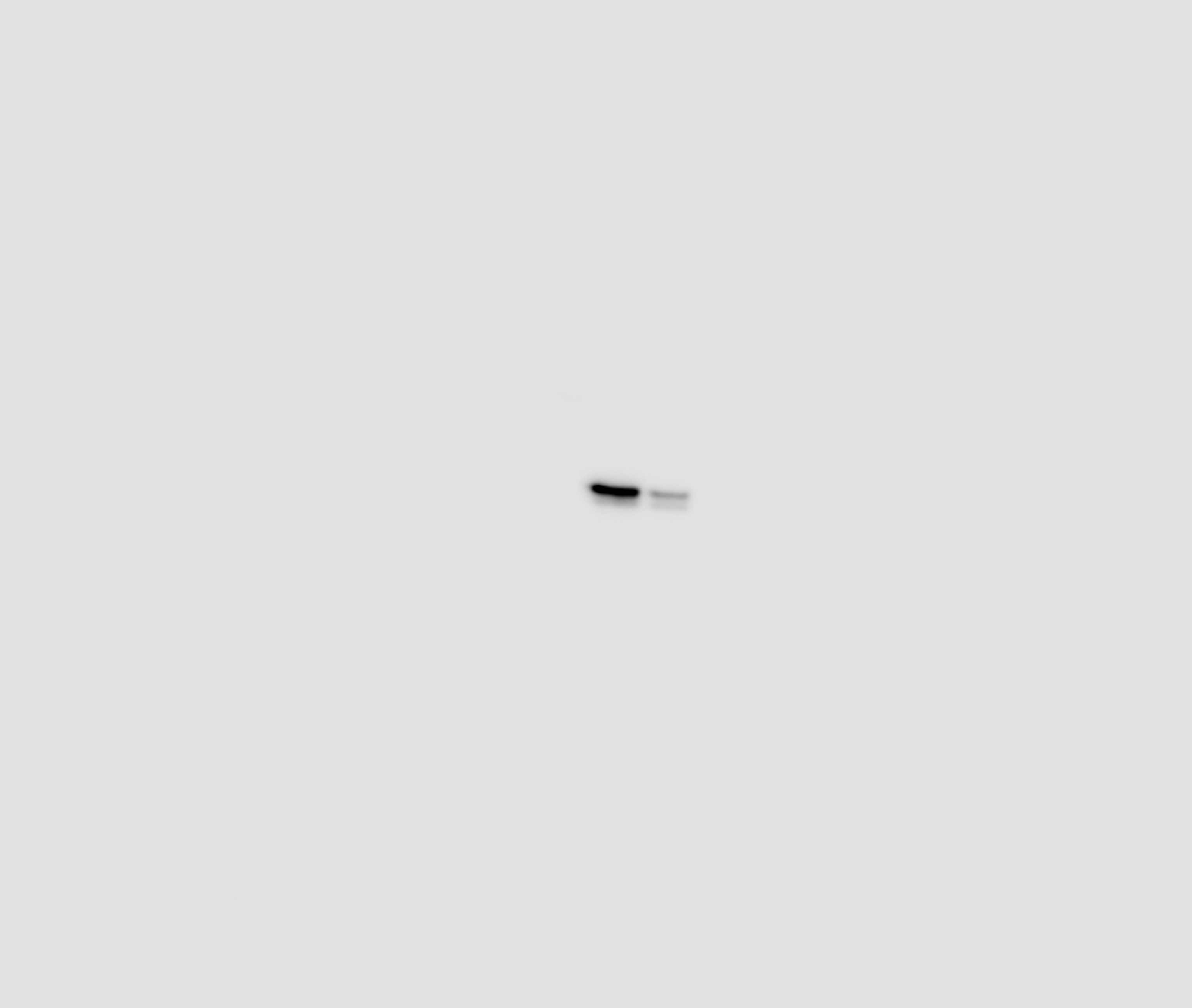

Supplement: Figure 2—source data 1. [file elife-76497-fig2-data1.zip › Figure 2-source data 1/Figure 2A-capzb.tif]

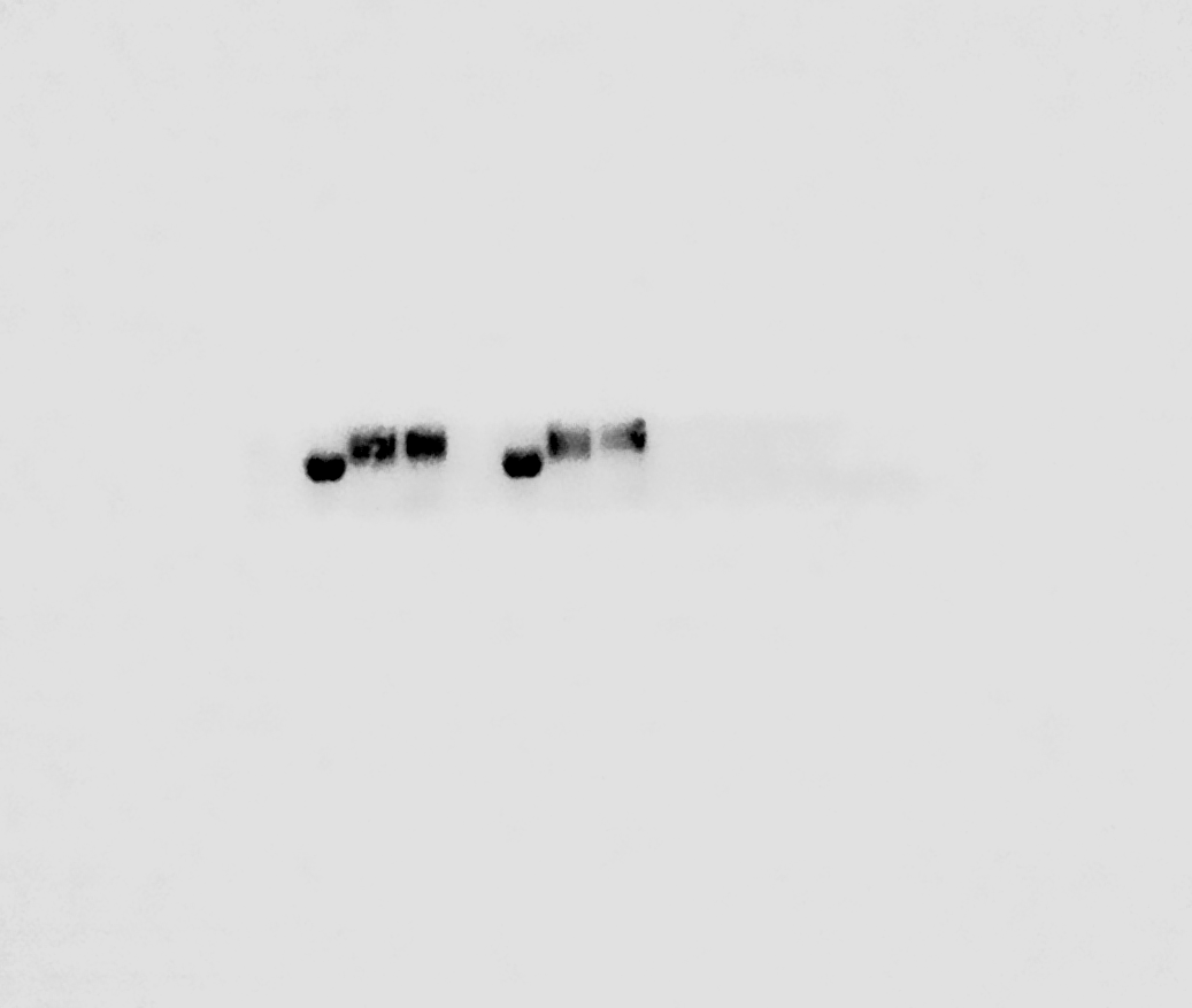

Supplement: Figure 2—source data 1. [file elife-76497-fig2-data1.zip › Figure 2-source data 1/Figure 2A-Samd14.tif]

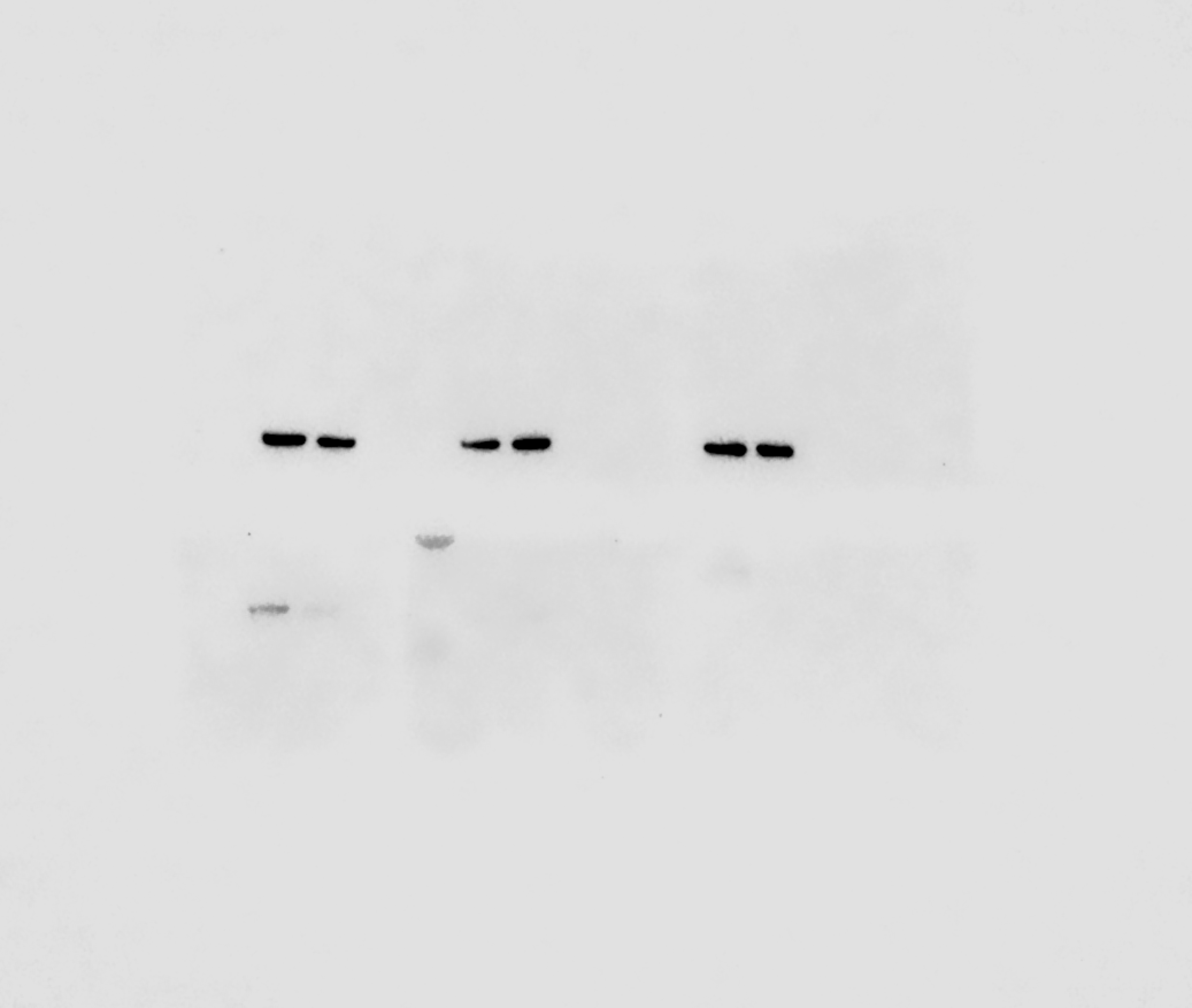

Supplement: Figure 3—source data 1. [file elife-76497-fig3-data1.zip › Figure 3-source data 1/Figure 3C- beta actin.tif]

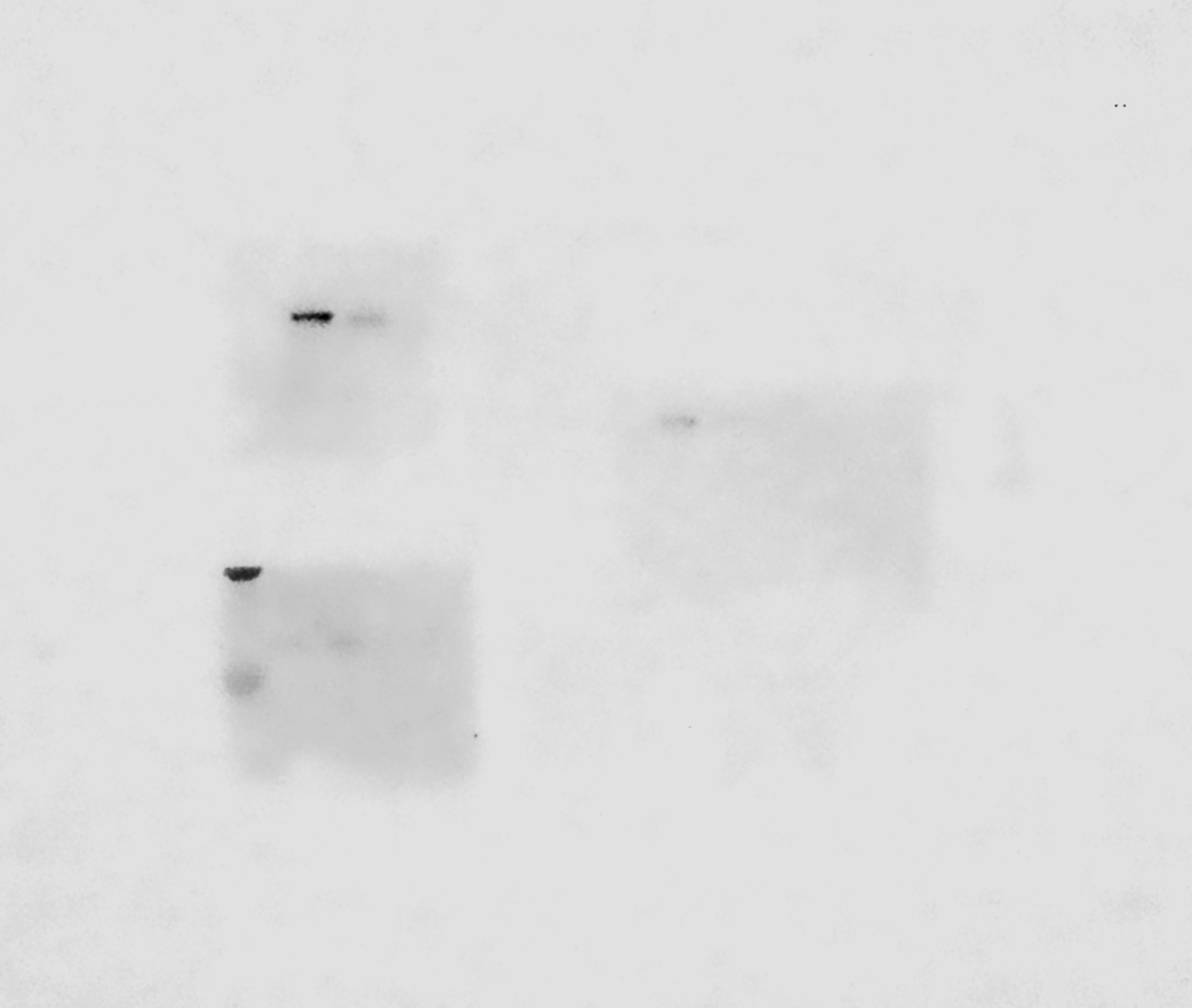

Supplement: Figure 3—source data 1. [file elife-76497-fig3-data1.zip › Figure 3-source data 1/Figure 3C- capzb.tif]

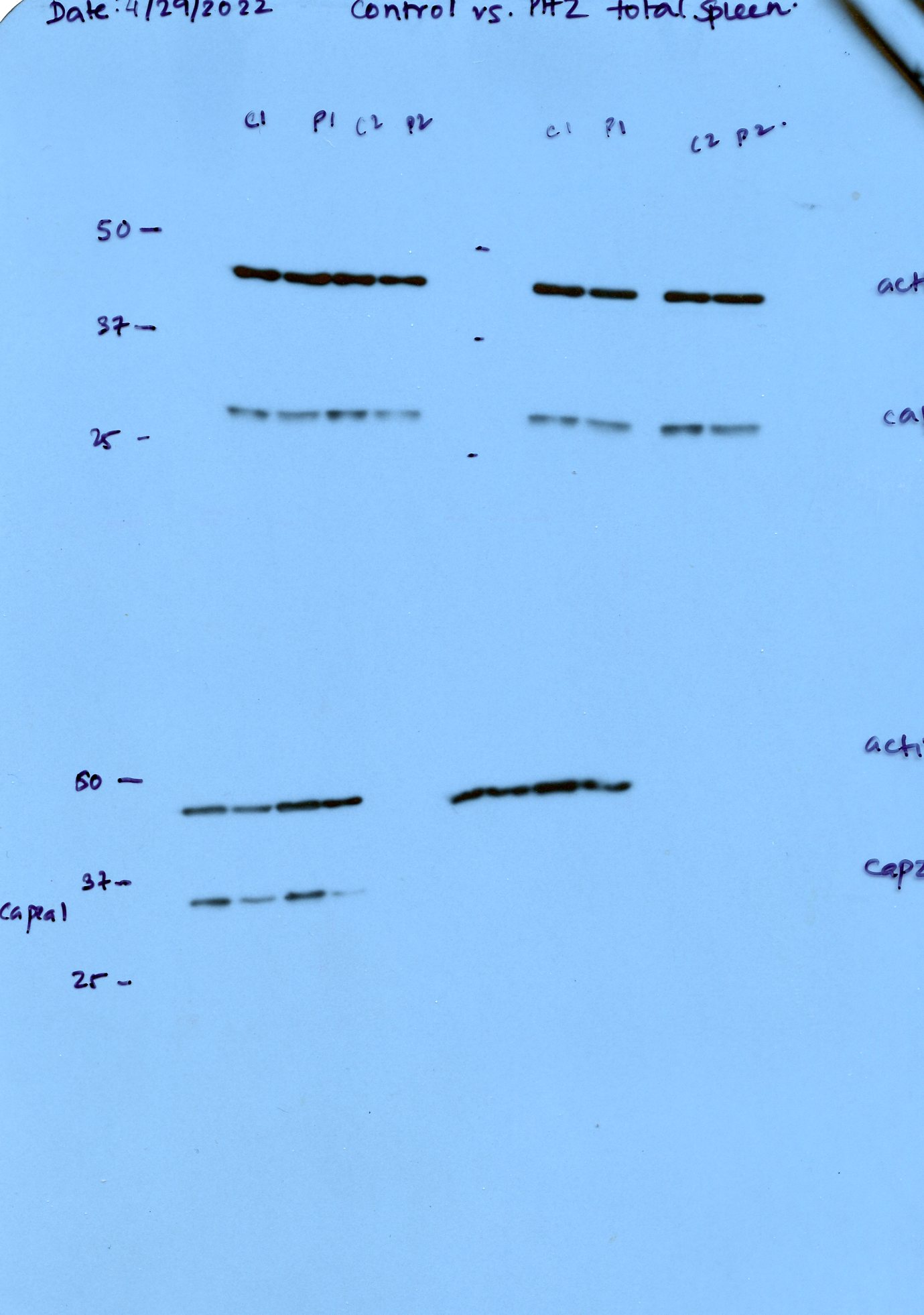

Supplement: Figure 3—figure supplement 1—source data 1. [file elife-76497-fig3-figsupp1-data1.zip › Figure 3-figure supplement 1-source data/Figure 3 Supplement 1-B.jpg]

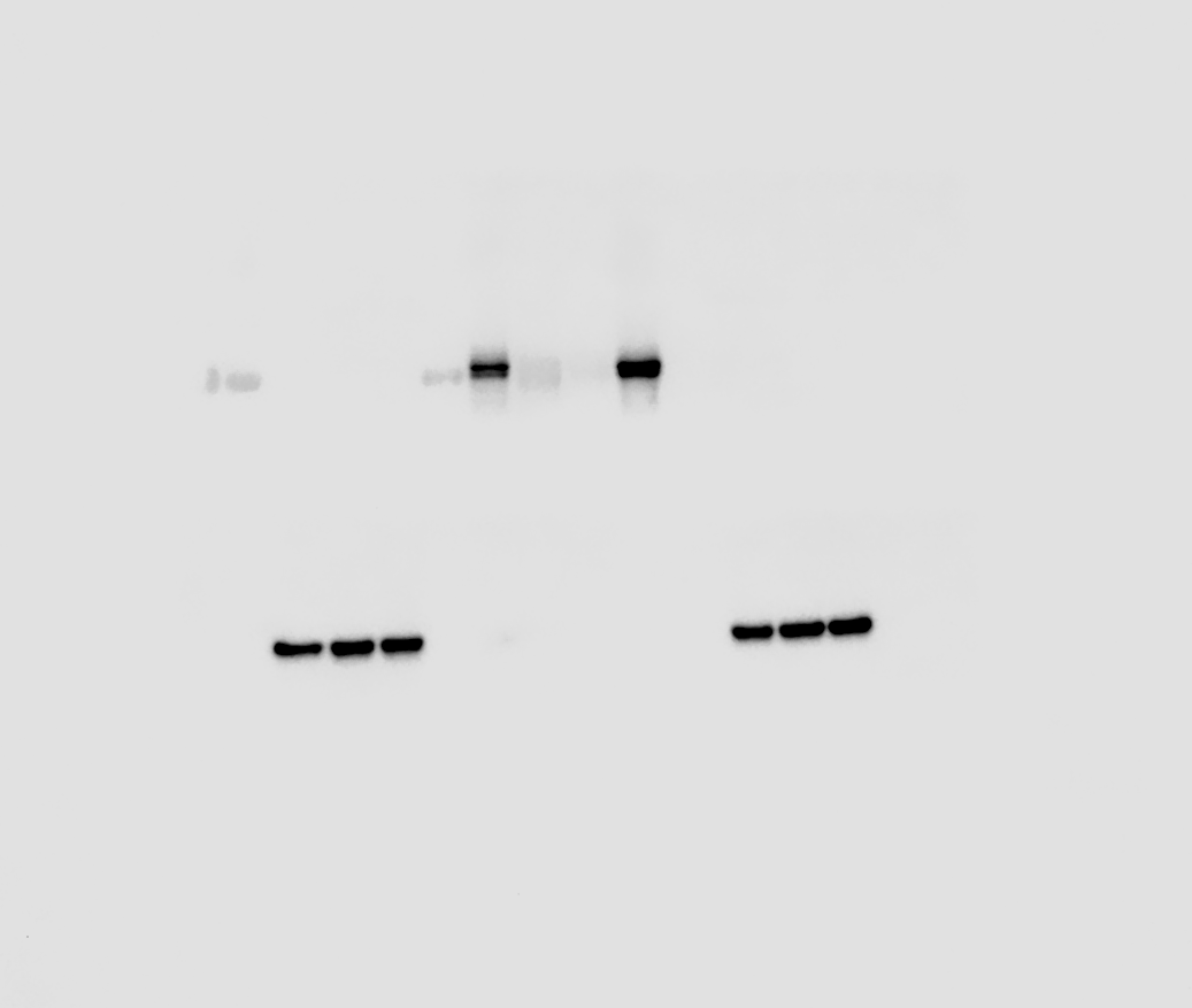

Supplement: Figure 4—source data 1. [file elife-76497-fig4-data1.zip › Figure 4-source data 1/Figure 4C-capzb input.tif]

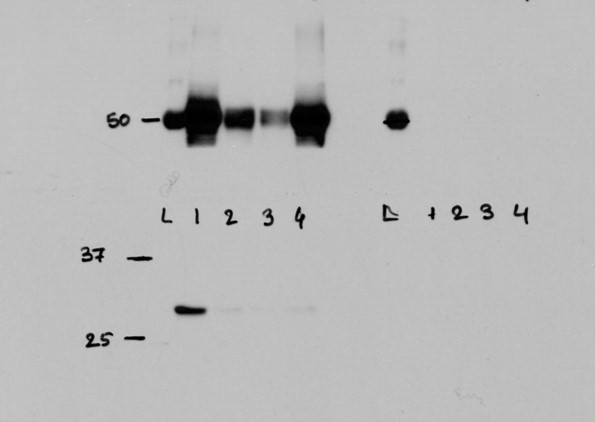

Supplement: Figure 4—source data 1. [file elife-76497-fig4-data1.zip › Figure 4-source data 1/Figure 4C-capzb IP.jpg]

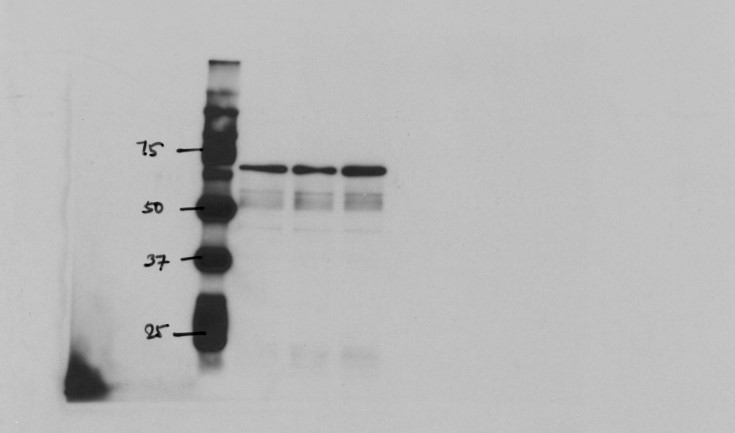

Supplement: Figure 4—source data 1. [file elife-76497-fig4-data1.zip › Figure 4-source data 1/Figure 4C-Samd14 Input.jpg]

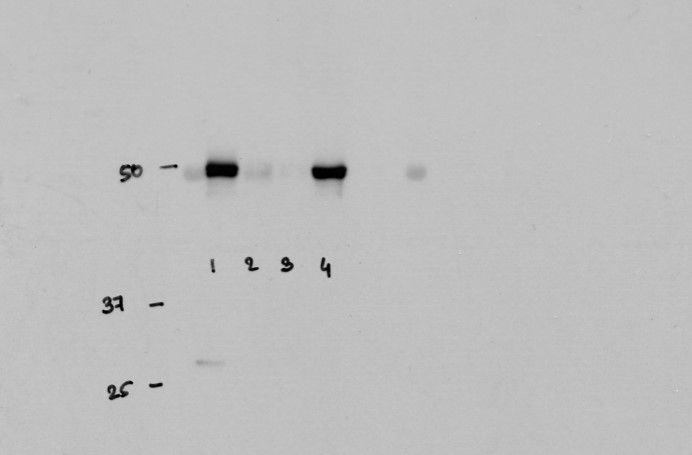

Supplement: Figure 4—source data 1. [file elife-76497-fig4-data1.zip › Figure 4-source data 1/Figure 4C-Samd14 IP.jpg]

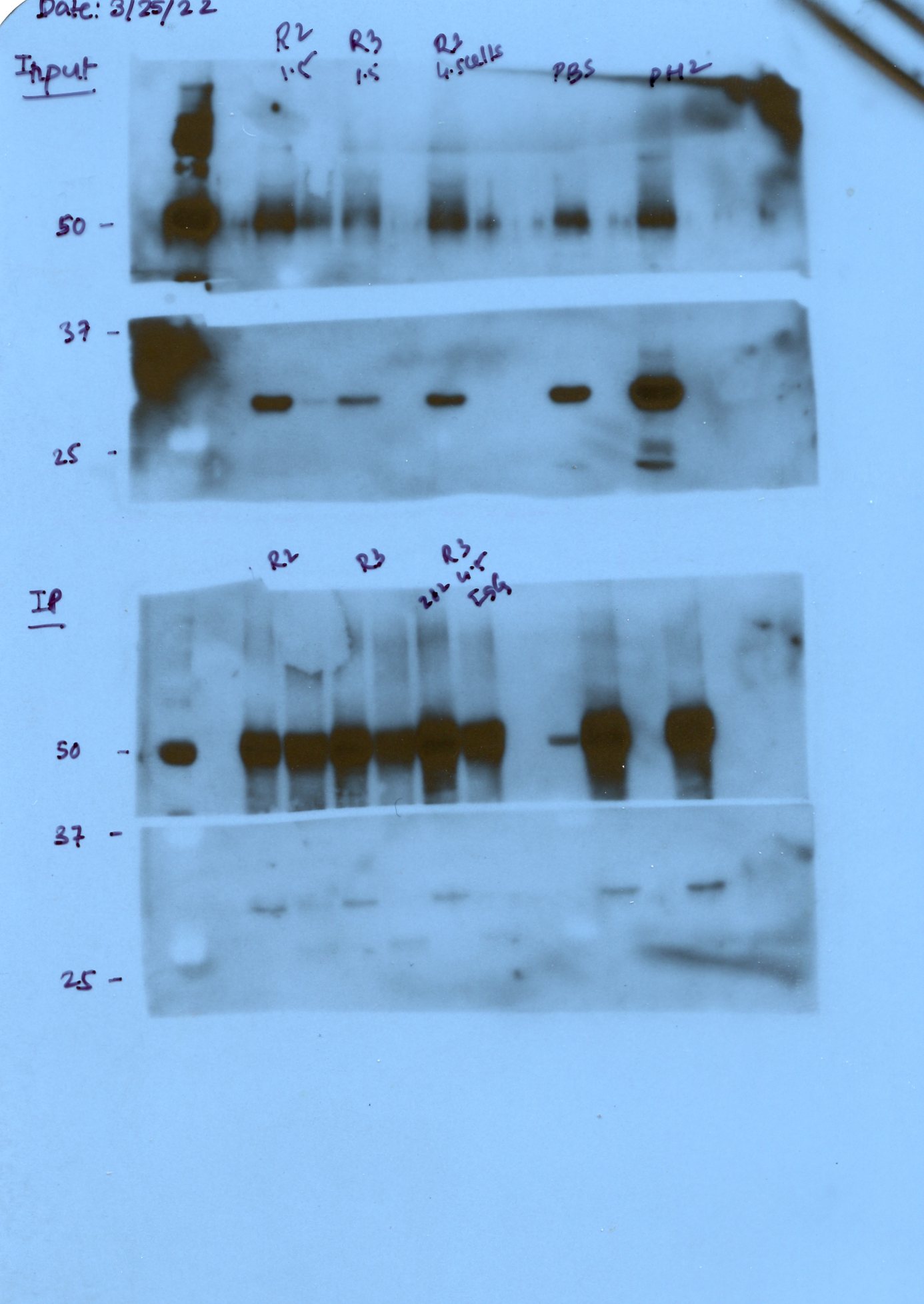

Supplement: Figure 4—figure supplement 1—source data 1. [file elife-76497-fig4-figsupp1-data1.zip › Figure 4-figure supplement1-source data/Figure 4 - Figure supplement 1-capzb IP.jpg]

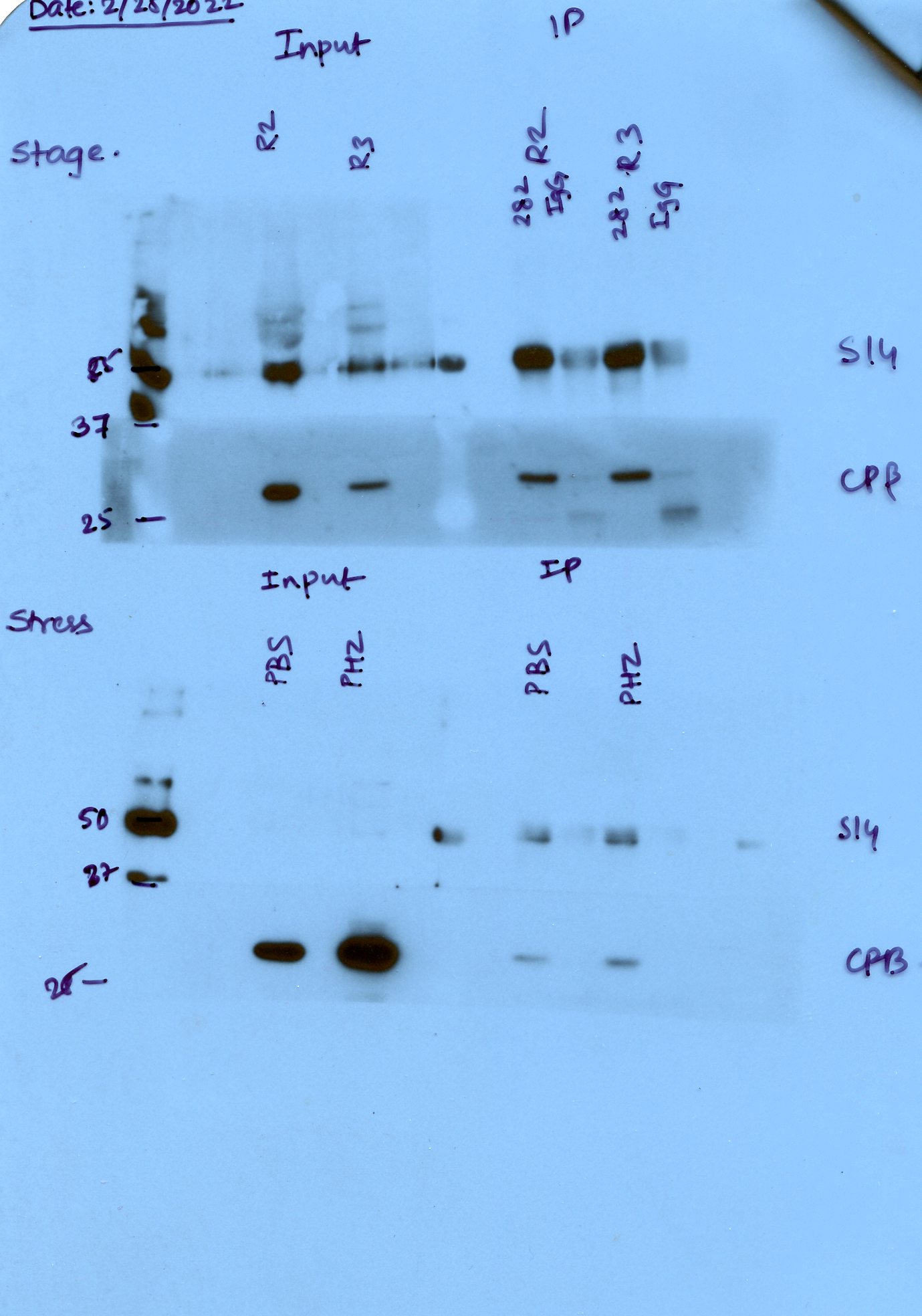

Supplement: Figure 4—figure supplement 1—source data 1. [file elife-76497-fig4-figsupp1-data1.zip › Figure 4-figure supplement1-source data/Figure 4-Figure supplement 1- Stress Samd14_IP and Capzb_input.jpg]

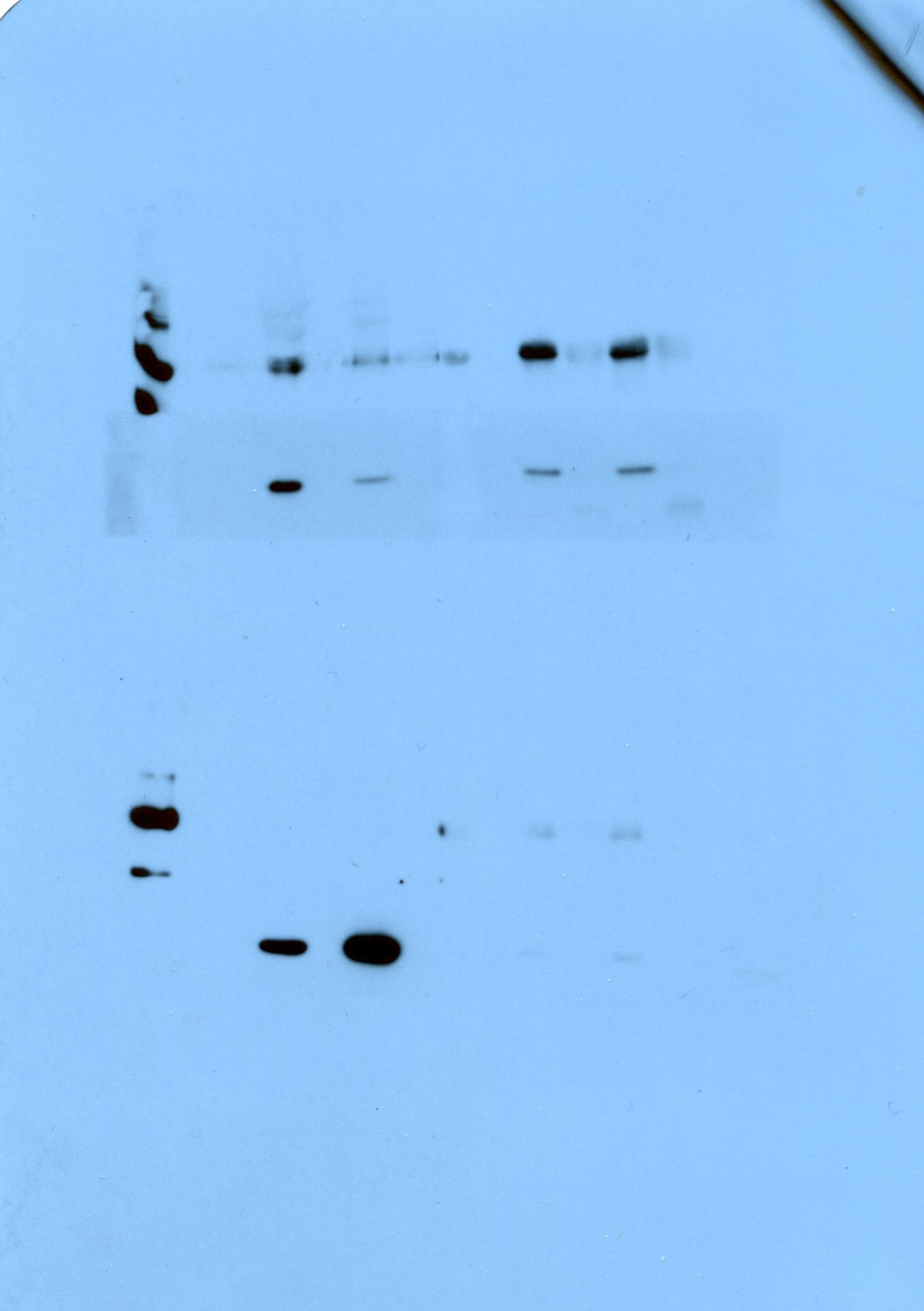

Supplement: Figure 4—figure supplement 1—source data 1. [file elife-76497-fig4-figsupp1-data1.zip › Figure 4-figure supplement1-source data/Figure 4-Figure supplement 1-3B-R2-R3.jpg]

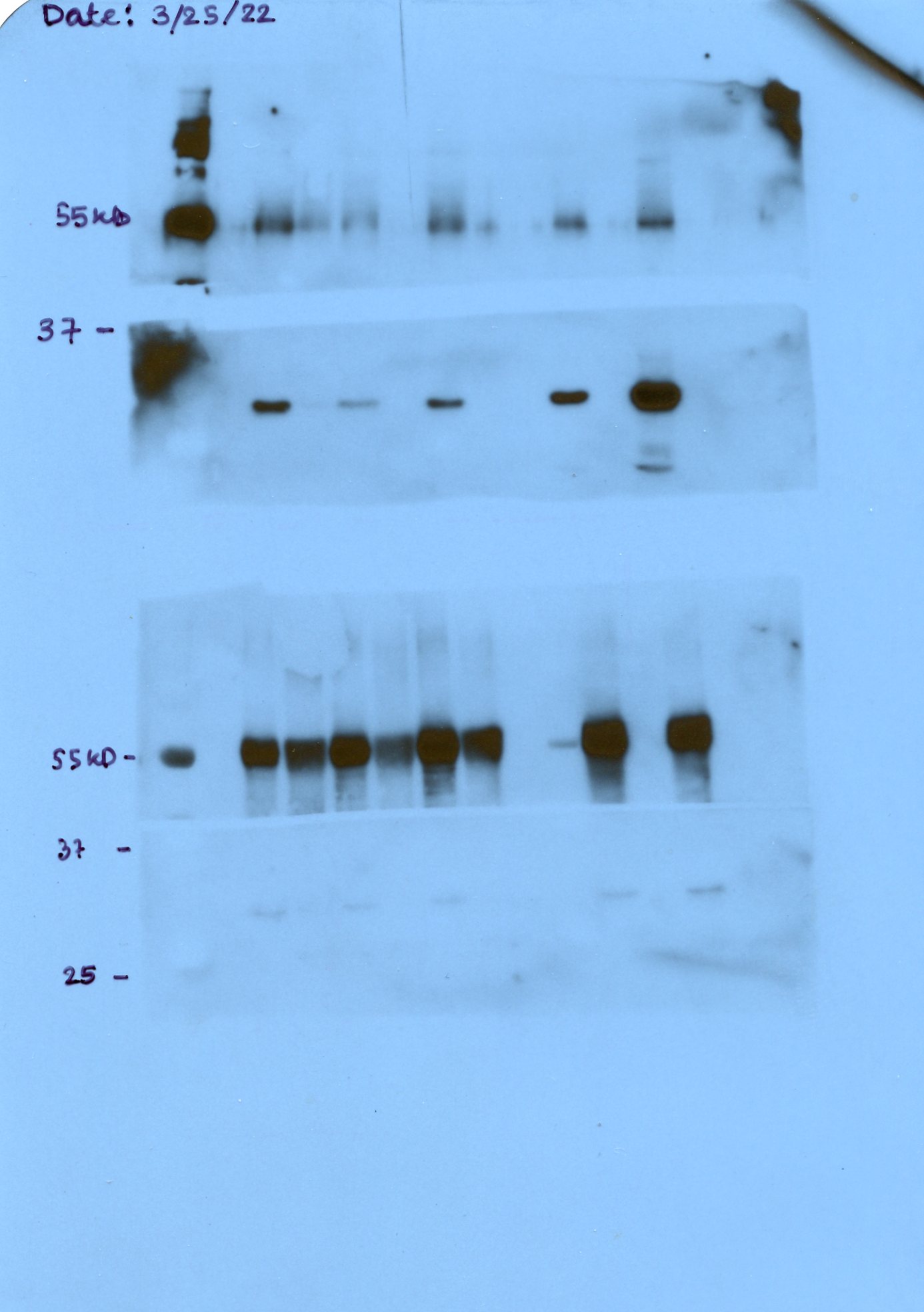

Supplement: Figure 4—figure supplement 1—source data 1. [file elife-76497-fig4-figsupp1-data1.zip › Figure 4-figure supplement1-source data/Figure 4-Figure supplement 1-Stress.jpg]

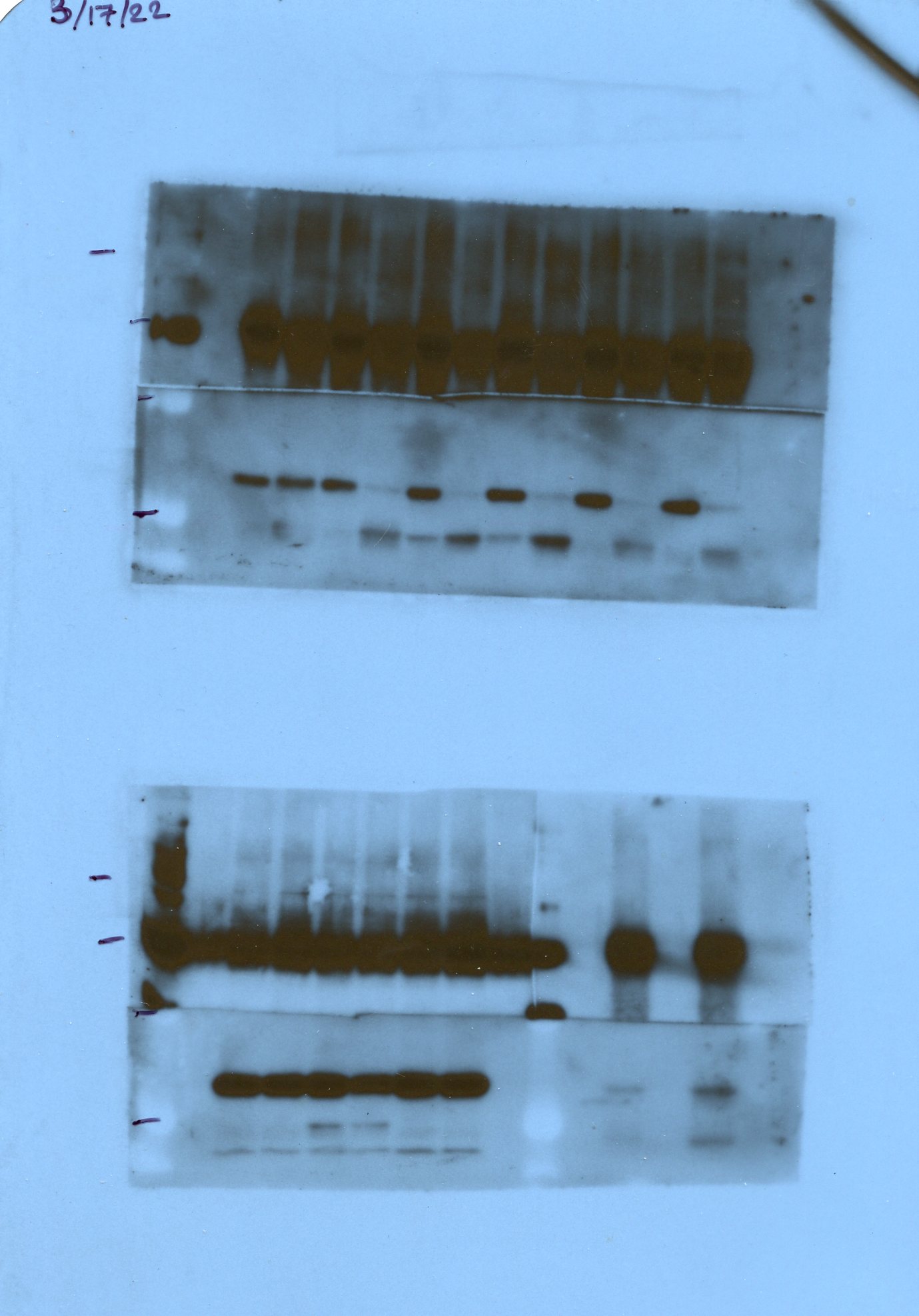

Supplement: Figure 4—figure supplement 1—source data 1. [file elife-76497-fig4-figsupp1-data1.zip › Figure 4-figure supplement1-source data/Supplement SCF- Capzb long exposure.jpg]

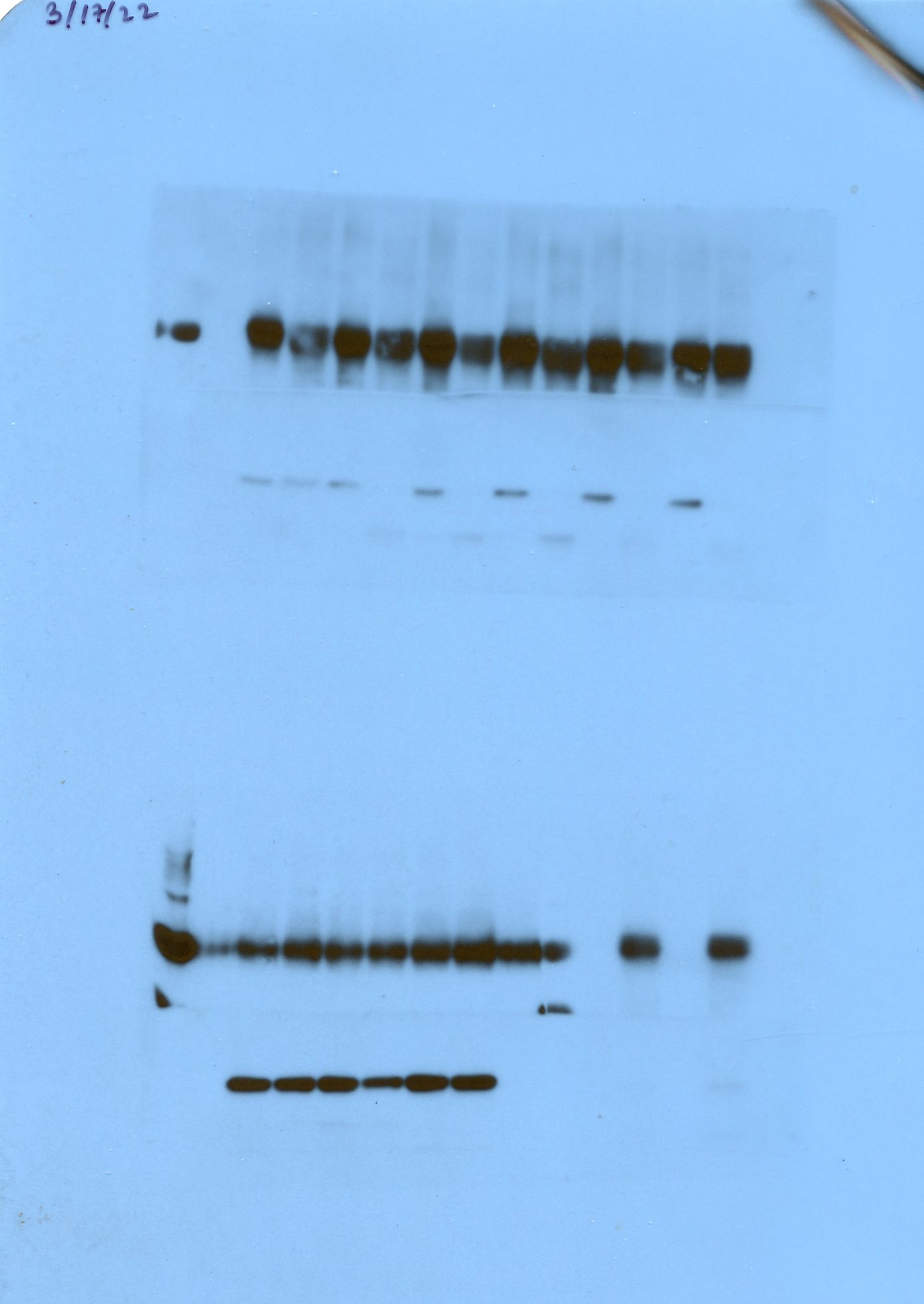

Supplement: Figure 4—figure supplement 1—source data 1. [file elife-76497-fig4-figsupp1-data1.zip › Figure 4-figure supplement1-source data/Supplement SCF-IP- Capzb short exposure.jpg]

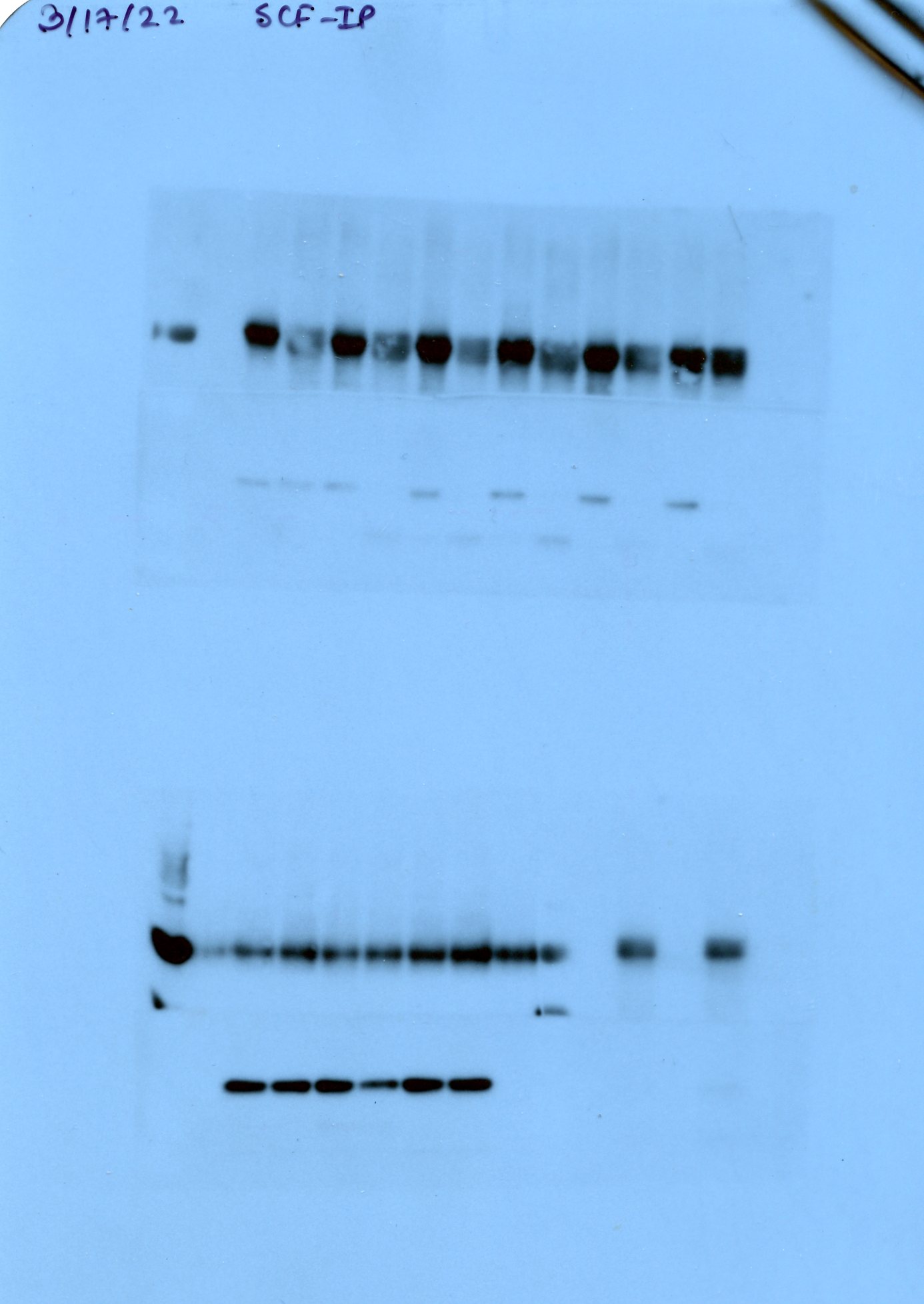

Supplement: Figure 4—figure supplement 1—source data 1. [file elife-76497-fig4-figsupp1-data1.zip › Figure 4-figure supplement1-source data/Supplement SCF-IP Inputs.jpg]

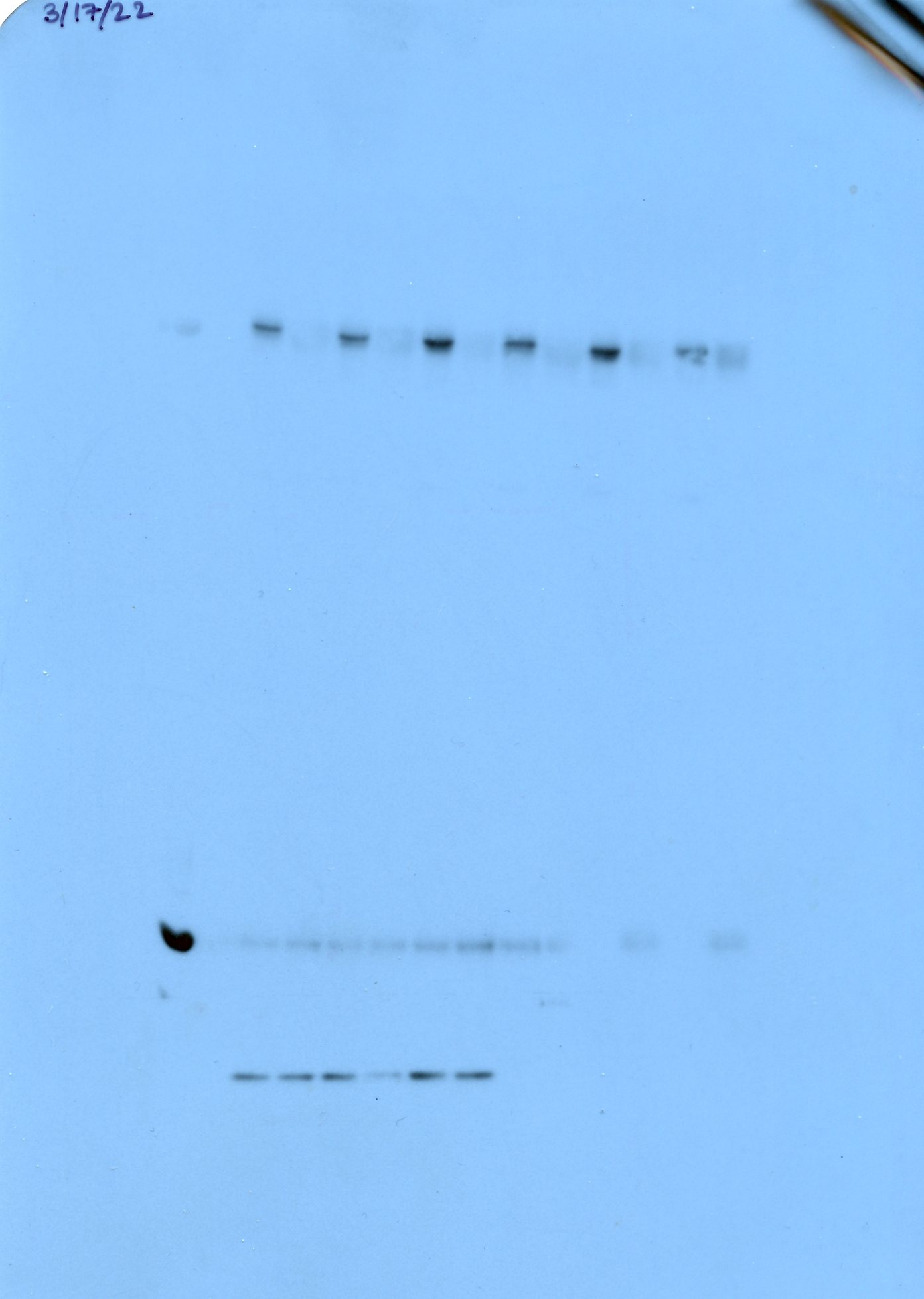

Supplement: Figure 4—figure supplement 1—source data 1. [file elife-76497-fig4-figsupp1-data1.zip › Figure 4-figure supplement1-source data/Supplement SCF-IP Samd14_IP.jpg]

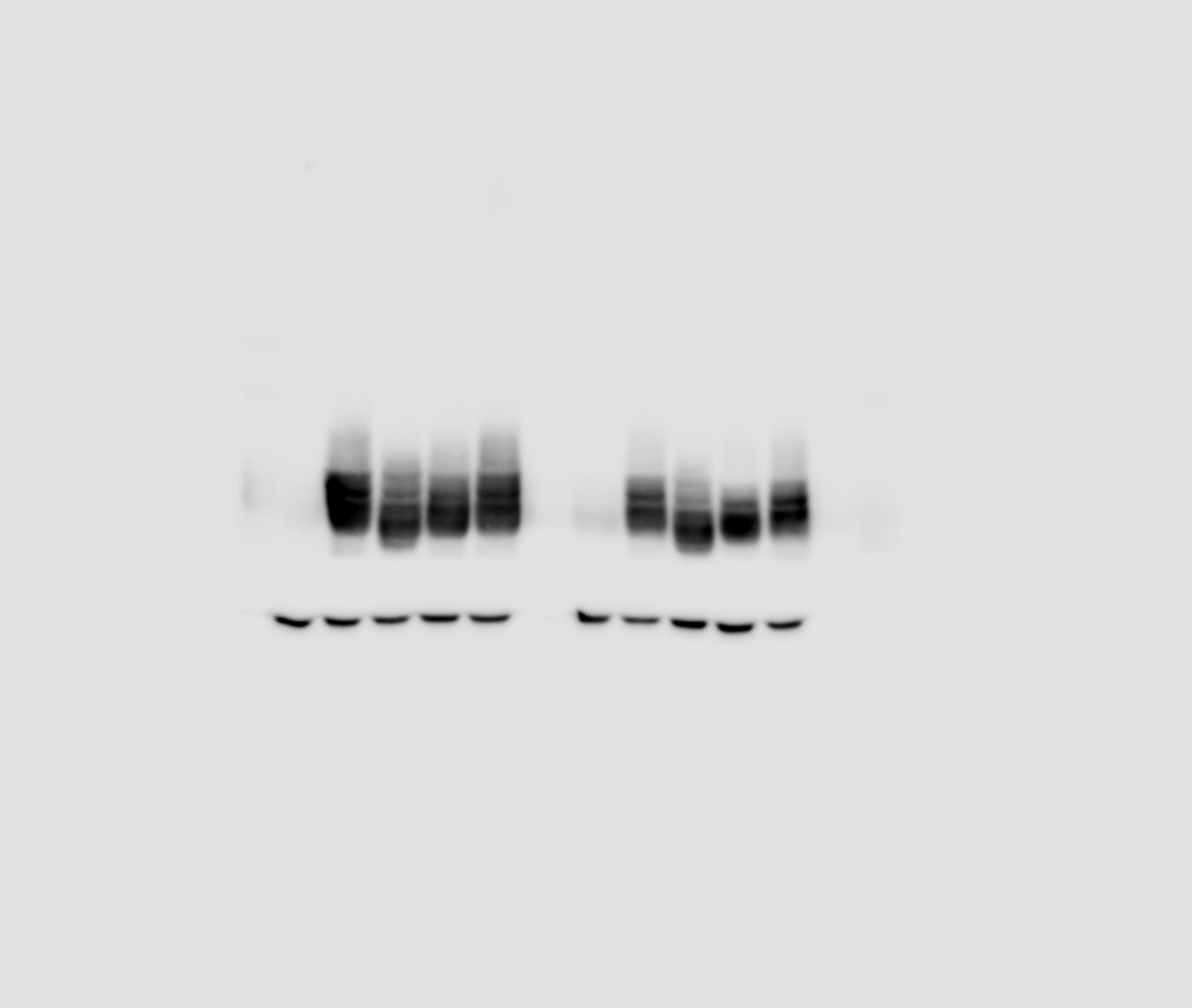

Supplement: Figure 6—source data 1. [file elife-76497-fig6-data1.zip › Figure 6-source data 1/Figure 6D-HA and beta actin.tif]

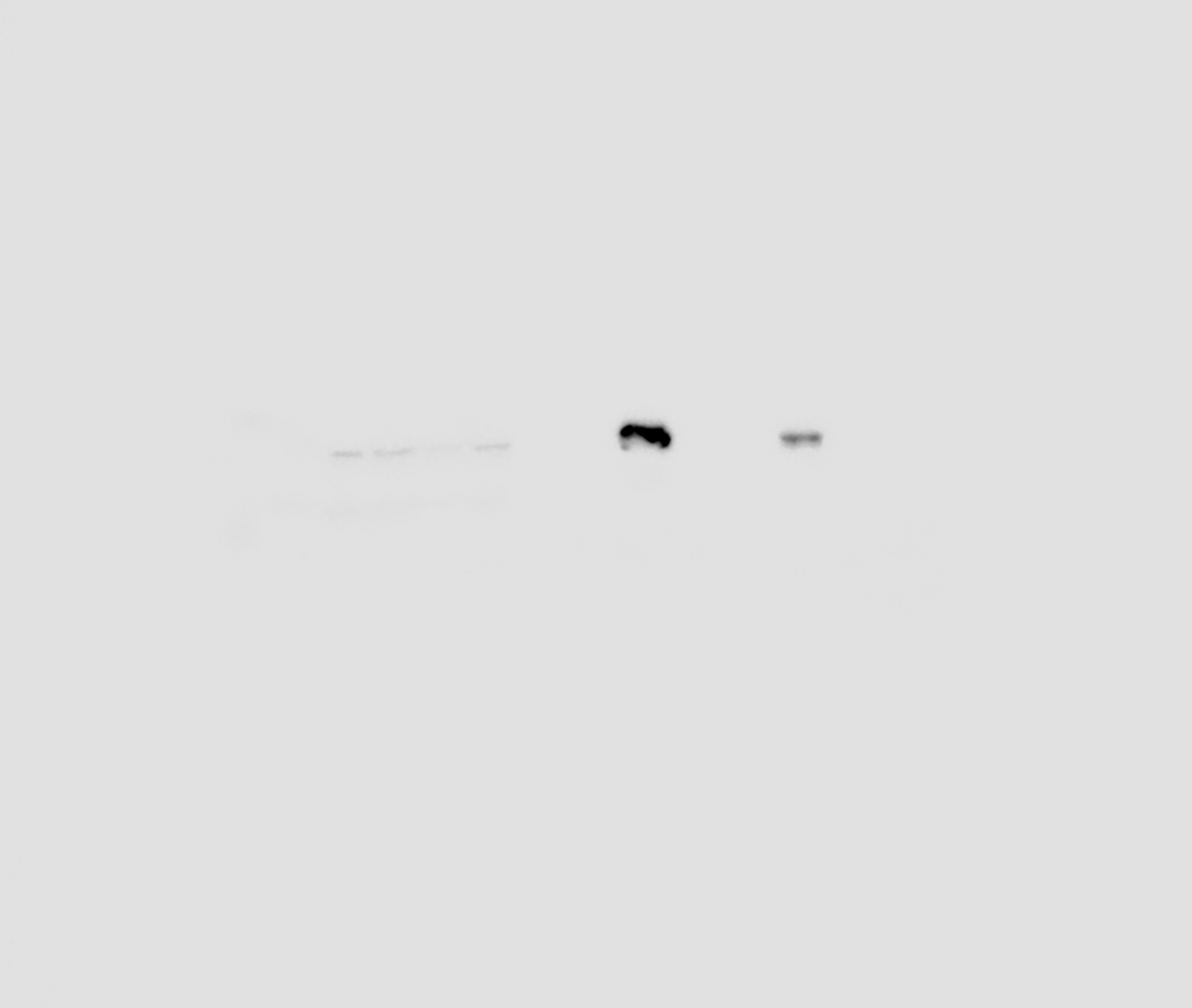

Supplement: Figure 6—source data 1. [file elife-76497-fig6-data1.zip › Figure 6-source data 1/Figure 6E-high GFP capza1.tif]

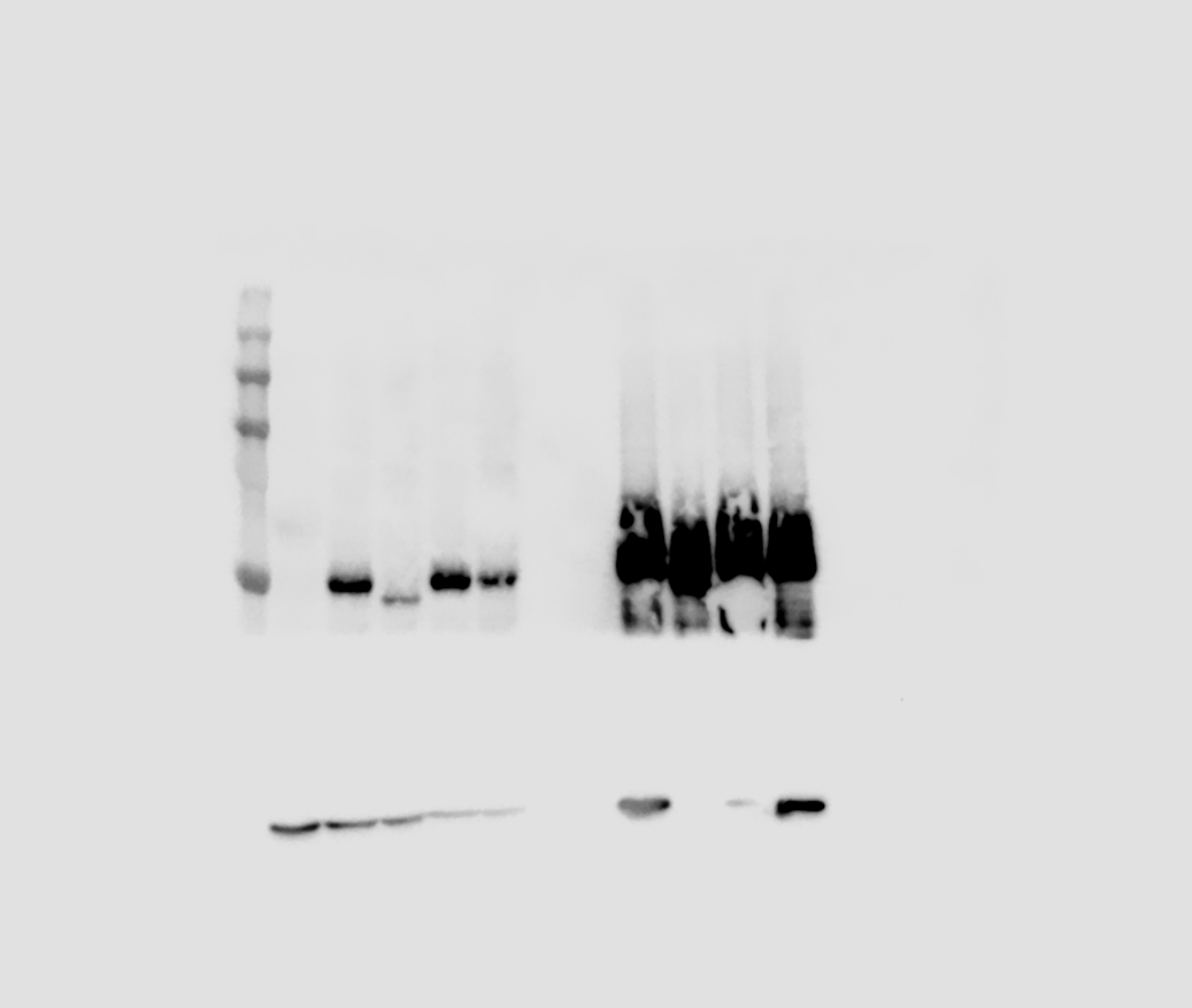

Supplement: Figure 6—source data 1. [file elife-76497-fig6-data1.zip › Figure 6-source data 1/Figure 6E-high GFP Capzb.tif]

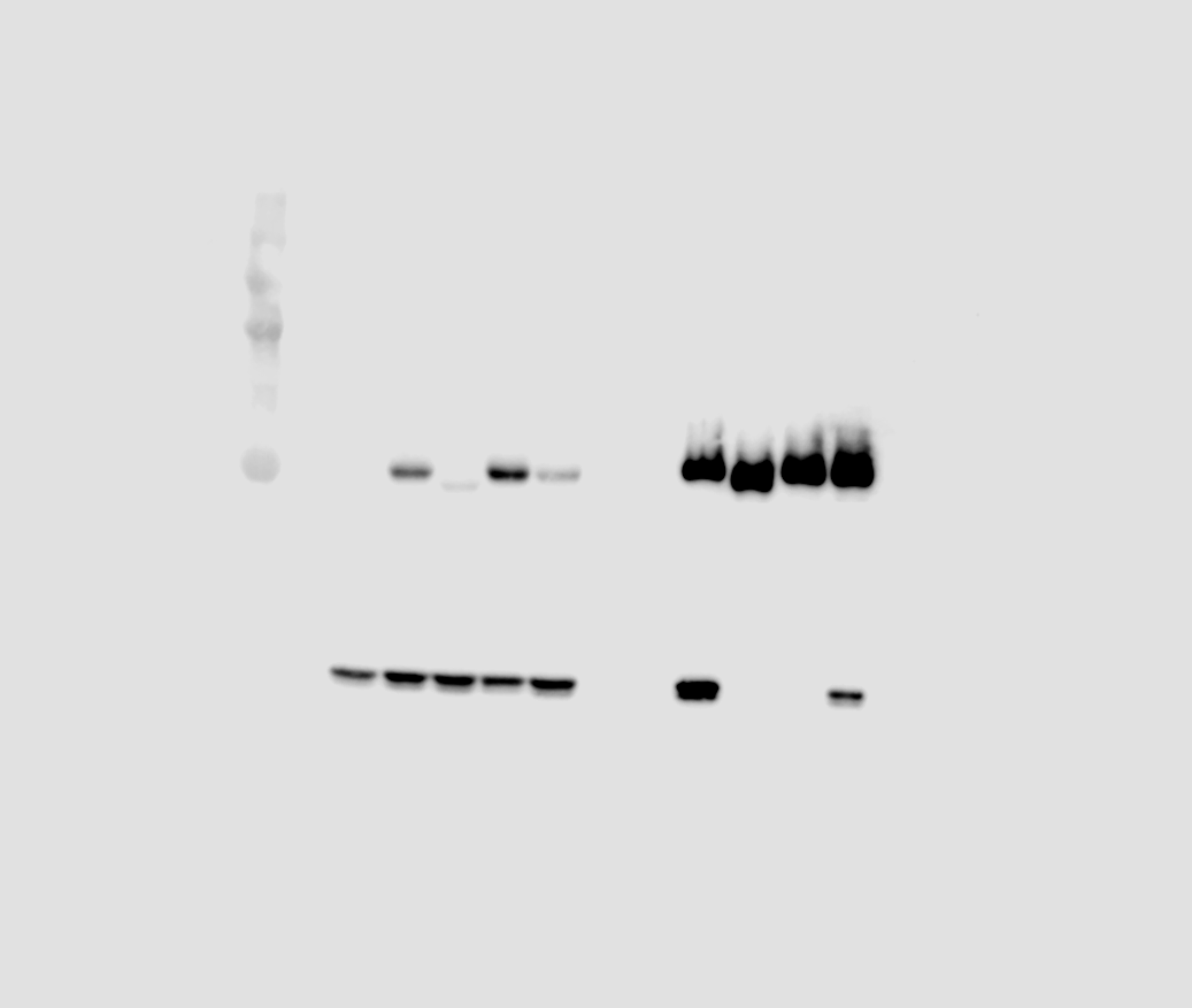

Supplement: Figure 6—source data 1. [file elife-76497-fig6-data1.zip › Figure 6-source data 1/Figure 6E-high GFP HA and Capza2.tif]

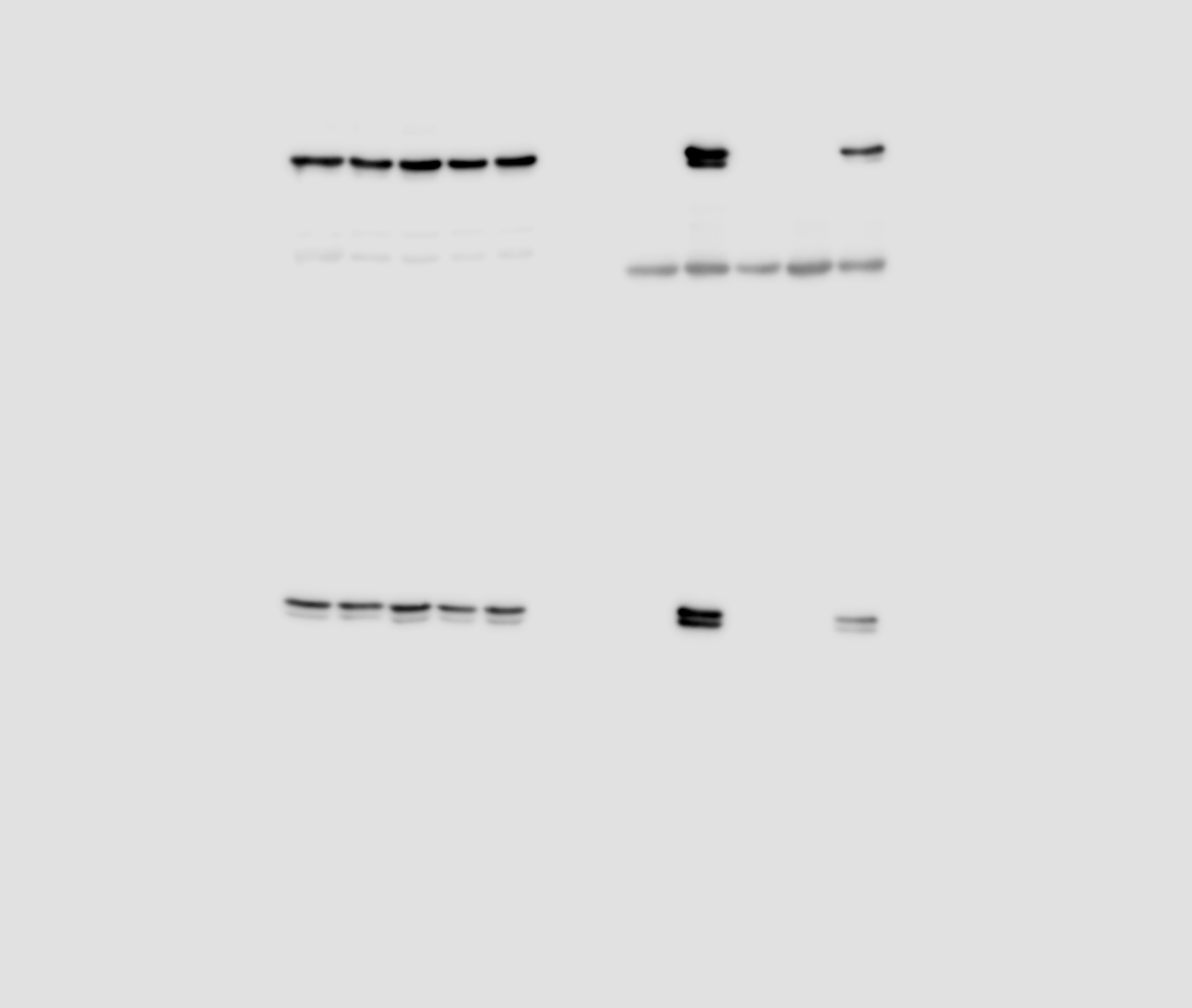

Supplement: Figure 6—source data 1. [file elife-76497-fig6-data1.zip › Figure 6-source data 1/Figure 6E-low GFP capza1 Capza2.tif]

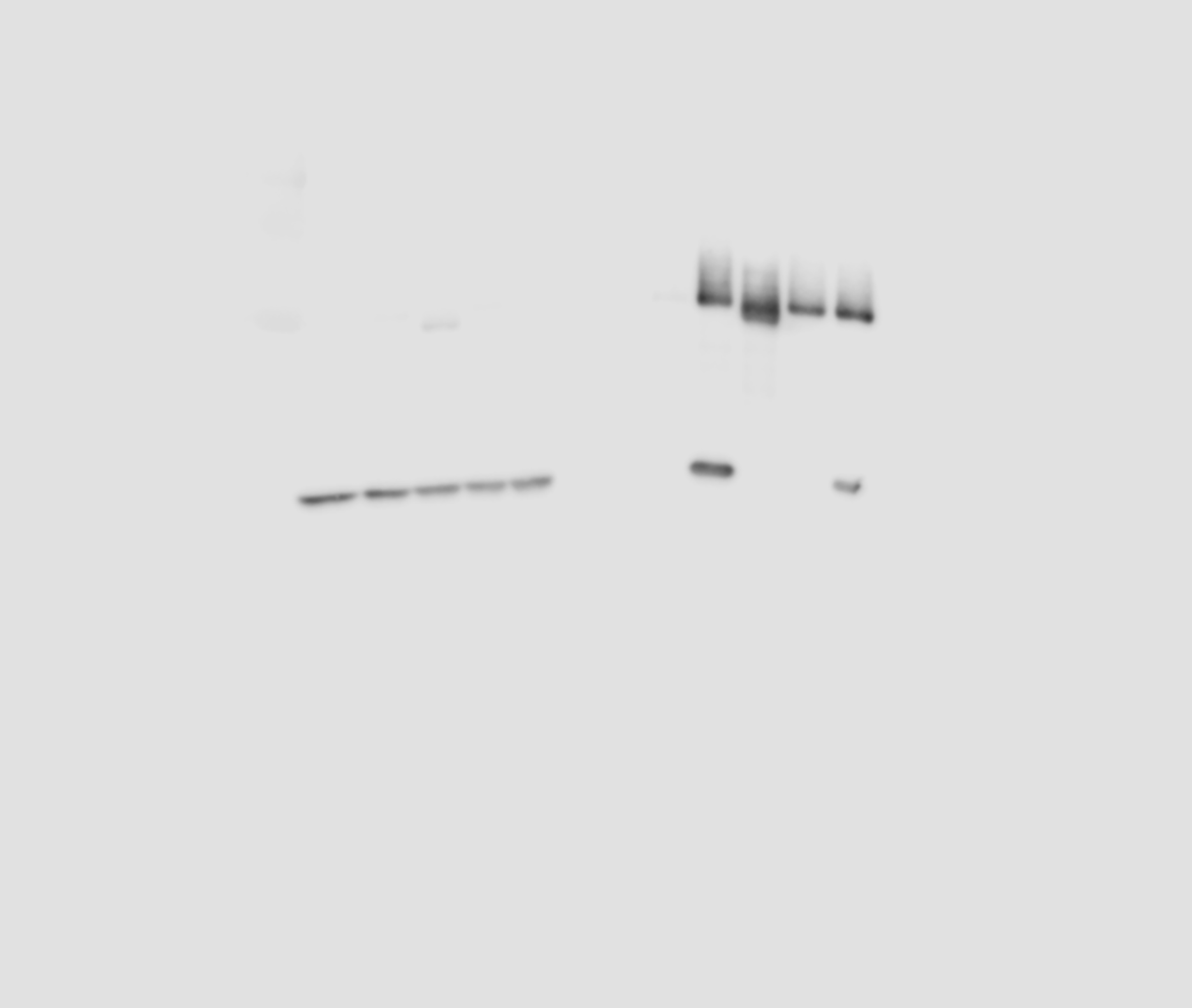

Supplement: Figure 6—source data 1. [file elife-76497-fig6-data1.zip › Figure 6-source data 1/Figure 6E-low GFP HA and Capzb.tif]
