# Supplementary material for: Functional requirements for a Samd14-capping protein complex in stress erythropoiesis
Source: eLife. 2022 Jun 17;11:e76497. doi: 10.7554/eLife.76497 (PMC9282853; doi:10.7554/eLife.76497)
Supplement: Transparent reporting form [file elife-76497-transrepform1.docx]

***eLife’s* transparent reporting form**

We encourage authors to provide detailed information *within their submission* to facilitate the interpretation and replication of experiments. Authors can upload supporting documentation to indicate the use of appropriate reporting guidelines for health-related research (see [EQUATOR Network](http://www.equator-network.org/%20)), life science research (see the [BioSharing Information Resource](https://biosharing.org/)), or the [ARRIVE guidelines](http://www.plosbiology.org/article/info:doi/10.1371/journal.pbio.1000412) for reporting work involving animal research. Where applicable, authors should refer to any relevant reporting standards documents in this form.

If you have any questions, please consult our Journal Policies and/or contact us: [editorial@elifesciences.org](mailto:editorial@elifesciences.org).

**Sample-size estimation**

- You should state whether an appropriate sample size was computed when the study was being designed
- You should state the statistical method of sample size computation and any required assumptions
- If no explicit power analysis was used, you should describe how you decided what sample (replicate) size (number) to use

Please outline where this information can be found within the submission (e.g., sections or figure legends), or explain why this information doesn’t apply to your submission:

1-2x10^7^ cells were used for IP-mass spectrometry (N=4) and immunoprecipitations with biological and technical duplicates on cells sorted based on 2 different GFP intensities to correct for potential ectopic protein-protein interactions based on expression levels. This information is in Supplemental Figure 1 and Methods sections. CFU assays were plated as biological triplicate and technically repeated twice (N=9). FACS analyses were with 50,000 cells per analysis consistent with standard lab protocols. This information is in the Figure legends and Methods section.

**Replicates**

- You should report how often each experiment was performed
- You should include a definition of biological versus technical replication
- The data obtained should be provided and sufficient information should be provided to indicate the number of independent biological and/or technical replicates
- If you encountered any outliers, you should describe how these were handled
- Criteria for exclusion/inclusion of data should be clearly stated
- High-throughput sequence data should be uploaded before submission, with a private link for reviewers provided (these are available from both GEO and ArrayExpress)

Please outline where this information can be found within the submission (e.g., sections or figure legends), or explain why this information doesn’t apply to your submission:

IP-mass spectrometry was conducted from 4 biological replicates and immunoprecipitations-Western blotting with biological and technical duplicates. The mass spectrometry proteomics data have been deposited to the ProteomeXchange Consortium via the PRIDE (Perez-Riverol et al, 2019) partner repository with the dataset identifier PXD030467 and 10.6019/PXD030467. This information is in the Methods sections and figure legends. CFU assays were plated as biological triplicate and technically repeated twice (N=9). We did encounter ‘outliers’, but could not establish a logical rationale for excluding them, so data presented in figures represents all data collected for each experiment. Quantitation of live cell FACS analyses were conducted on at least three replicates, consistent with standard lab protocols. FACS analyses for phospho-flow cytometry were an average of at least 6 and up to 12 individual replicates, depending on the cellular material collected from mouse spleen and the number of comparisons conducted in each experiment. The specific number of replicates is in figure legends and individual data points are shown in each associated figure.

**Statistical reporting**

- Statistical analysis methods should be described and justified
- Raw data should be presented in figures whenever informative to do so (typically when N per group is less than 10)
- For each experiment, you should identify the statistical tests used, exact values of N, definitions of center, methods of multiple test correction, and dispersion and precision measures (e.g., mean, median, SD, SEM, confidence intervals; and, for the major substantive results, a measure of effect size (e.g., Pearson's r, Cohen's d)
- Report exact p-values wherever possible alongside the summary statistics and 95% confidence intervals. These should be reported for all key questions and not only when the p-value is less than 0.05.

Please outline where this information can be found within the submission (e.g., sections or figure legends), or explain why this information doesn’t apply to your submission:

Statistics are presented as Mean +/- SD, detailed in the methods, and confidence intervals are indicated in each figure and figure legends as *p < 0.05; **p < 0.01; ***p < 0.001; ****p < 0.0001. Statistics for mass spectrometry was conducted using Scaffold (Proteome Software; v. 4.8.9) using a 99.0% protein probability with a minimum of 2 unique peptides with at least 80% peptide probability. Statistically enriched proteins were determined using a 2-way ANOVA. These methods and analyses are included in Methods section

(For large datasets, or papers with a very large number of statistical tests, you may upload a single table file with tests, Ns, etc., with reference to sections in the manuscript.)

**Group allocation**

- Indicate how samples were allocated into experimental groups (in the case of clinical studies, please specify allocation to treatment method); if randomization was used, please also state if restricted randomization was applied
- Indicate if masking was used during group allocation, data collection and/or data analysis

Please outline where this information can be found within the submission (e.g., sections or figure legends), or explain why this information doesn’t apply to your submission:

Pooled cells from a single mouse were separately infected with each expression construct and cultured separately until experimental endpoints. These are described in Methods. Identically treated samples were grouped for analysis based on expression construct and treatment method. Individual biological replicates for each condition were analyzed together before moving on to the next replicate, to avoid batch effects.

**Additional data files (“source data”)**

- We encourage you to upload relevant additional data files, such as numerical data that are represented as a graph in a figure, or as a summary table
- Where provided, these should be in the most useful format, and they can be uploaded as “Source data” files linked to a main figure or table
- Include model definition files including the full list of parameters used
- Include code used for data analysis (e.g., R, MatLab)
- Avoid stating that data files are “available upon request”

Please indicate the figures or tables for which source data files have been provided:

Source data for Western blotting is provided in Supplemental Figure 2 and within source data files associated with each figure containing Western blots.
